# Label-Free Quantitative Analysis of Pig Liver Proteome after Hepatitis E Virus Infection

**DOI:** 10.3390/v16030408

**Published:** 2024-03-06

**Authors:** Camillo Martino, Alessio Di Luca, Francesca Bennato, Andrea Ianni, Fabrizio Passamonti, Elisa Rampacci, Michael Henry, Paula Meleady, Giuseppe Martino

**Affiliations:** 1 Department of Veterinary Medicine, University of Perugia, 06126 Perugia, Italy; camillo.martino@studenti.unipg.it (C.M.); fabrizio.passamonti@unipg.it (F.P.); elisa.rampacci@unipg.it (E.R.); 2 Department of BioScience and Technology for Food, Agriculture, and Environment, University of Teramo, 64100 Teramo, Italy; alessiodiluca@gmail.com (A.D.L.); fbennato@unite.it (F.B.); gmartino@unite.it (G.M.); 3 National Institute for Cellular Biotechnology, Dublin City University, D09 DX63 Dublin, Ireland; michael.henry@dcu.ie (M.H.); paula.meleady@dcu.ie (P.M.)

**Keywords:** hepatitis E virus, pig liver, proteomics, label-free quantification

## Abstract

Hepatitis E represents an emerging zoonotic disease caused by the Hepatitis E virus (HEV), for which the main route of transmission is foodborne. In particular, infection in humans has been associated with the consumption of contaminated undercooked meat of pig origin. The aim of this study was to apply comparative proteomics to determine if porcine liver protein profiles could be used to distinguish between pigs seropositive and seronegative for HEV. Preliminarily, an ELISA was used to evaluate the presence of anti-HEV antibodies in the blood serum of 136 animals sent to slaughter. Among the analyzed samples, a seroprevalence of 72.8% was estimated, and it was also possible to identify 10 animals, 5 positive and 5 negative, coming from the same farm. This condition created the basis for the quantitative proteomics comparison between homogeneous animals, in which only the contact with HEV should represent the discriminating factor. The analysis of the proteome in all samples of liver exudate led to the identification of 554 proteins differentially expressed between the two experimental groups, with 293 proteins having greater abundance in positive samples and 261 more represented in negative exudates. The pathway enrichment analysis allowed us to highlight the effect of the interaction between HEV and the host biological system in inducing the potential enrichment of 69 pathways. Among these, carbon metabolism stands out with the involvement of 41 proteins, which were subjected to interactomic analysis. This approach allowed us to focus our attention on three enzymes involved in glycolysis: glucose-6-phosphate isomerase (GPI), glyceraldehyde-3-phosphate dehydrogenase (GAPDH), and fructose-bisphosphate aldolase A (ALDOA). It therefore appears that infection with HEV induced a strengthening of the process, which involves the breakdown of glucose to obtain energy and carbon residues useful for the virus’s survival. In conclusion, the label-free LC-MS/MS approach showed effectiveness in highlighting the main differences induced on the porcine liver proteome by the interaction with HEV, providing crucial information in identifying a viral signature on the host metabolism.

## 1. Introduction

Hepatitis E represents an acute pathological disease with a worldwide distribution [1]. The biological agent responsible for this disease is represented by the hepatitis E virus (HEV), a small nonenveloped virus with a single-stranded RNA genome of 7.2 kb in length, containing three major open reading frames (ORF1, ORF2, and ORF3) responsible for the expression of structural and nonstructural proteins [2]. The virus is characterized by two main routes of infection [3]; large waterborne outbreaks generally accompany virus transmission in developing countries, whereas in industrialized countries, transmission is more associated with small outbreaks and it is considered a sporadic foodborne infection. These different epidemiological manifestations find justification in the fact that there are different genotypes of the virus [4]; genotypes 1 (HEV-1) and 2 (HEV-2) are generally responsible for the first event, while genotypes 3 (HEV-3) and 4 (HEV-4) commonly oversee the second. With reference to this aspect, it is important to underline that HEV-1 and HEV-2 have the possibility to infect only humans, while HEV-3 and HEV-4 represent zoonotic agents since they are able to infect domesticated animals, which therefore represent the main reservoirs in the transmission of the infection to humans. Besides this, it is also important to specify that HEV-4 is considered endemic in the Asian region, while HEV-3 is characterized by a global circulation [5].

In Western countries, especially those in Europe, the transmission of HEV in humans seems to be mainly attributed to the consumption by the population of undercooked pork and wild boar meat; the problem therefore concerns preparations that do not require heat treatments such as cured meats and sausages, especially if they contain the animal’s liver [6]. This mechanism of transmission in humans has been validated over time by various studies, including virological analyzes which have identified the same viral RNA sequence both in patients and in the consumed food, as well as epidemiological studies which have shown a strong statistical association between the consumption of foods containing pork liver and the risk of infection by HEV genotypes 3 and 4 [7]. With strict reference to pigs, the onset of susceptibility to viral infection occurs around 3–4 months of life because of the loss of maternal immunity [8]. The preferential transmission route for these animals is the fecal–oral one, which is followed by viremia, which involves the replication of the virus in the liver and its release into the environment through the feces; from an immunological point of view, the production of type M immunoglobulins (IgM) can be initially observed followed by an adaptive immune response mediated by type G immunoglobulins (IgG) in the terminal phase [9].

Another aspect of great interest is the identification of markers that may be representative of an ongoing infection or of an infection that has occurred. From this point of view, it should be considered that viral infections usually alter host cell functions, thus determining variations which, in most cases, can be well observed in the differential expression of specific proteins or groups of them. The improvement in methods for proteomic investigation has made it possible to investigate in an increasingly effective way the changes in cellular protein expression as a consequence of different stimuli; for example, this strategy has been applied to characterize the effects induced by different viruses, such as type-1 human immunodeficiency virus (HIV-1) and severe acute respiratory syndrome coronavirus (SARS-CoV) [10,11]. With specific regard to HEV infection, a proteomic study was conducted on the porcine liver, identifying 10 proteins potentially associated with the pathogenesis, with effects especially at the level of the expression of apolipoprotein E (ApoE) and ferritin heavy chain [12]. Similarly, Rogée et al. [13] attempted to characterize the changes induced in the porcine liver proteome during infection with three different strains of HEV genotype 3. The results highlighted the ability of the three HEV strains to influence several cellular processes; however, few differences were observed between the three strains, suggesting that viral genetic variability may be responsible for variations in the pathogenesis course.

In the present study, the objective is therefore to apply the tool of comparative proteomic investigation in identifying a characteristic signature at the level of the porcine liver following HEV infection. The rationale behind this research lies in the desire to confirm that the variations in the proteome induced by the virus could persist even following infection, therefore laying the foundations for the possibility of identifying useful markers to define the occurred contact between the pathogen and the host in animals that arrive at the slaughterhouse apparently in good condition.

## 2. Materials and Methods

Animals were slaughtered in commercial abattoirs of the Abruzzo region (Italy) in accordance with the European Council Regulation 1099/2009 [14] dealing on the protection of animals at the time of killing. All the activities involving animals were performed according to the European legislations (Directive 2010/63/EU) and no animals were slaughtered for the purpose of this study; therefore, for this work, ethical approval is not required under Italian law (Legislative Decree 26/2014) [15,16].

### 2.1. Sample Collection and Experimental Plan

In the period between April 2022 and June 2022, whole-blood and liver samples were collected from pigs slaughtered in the Abruzzo region (Italy). Overall, the sampling involved 136 Large White pigs from 12 farms of the region; each animal was killed upon reaching about 7 months of age and a body weight in the range of 96–108 kg. Both blood and liver tissue samples were transported to the laboratory within 2 h of the animal slaughtering, inside hermetically sealed containers at a controlled temperature of 8 °C. The blood samples were then centrifuged at 4000× *g* for 10 min to obtain the separation of serum that was collected into 1.5 mL clean, dried Eppendorf tubes (Eppendorf AG, Hamburg, Germany) and frozen at −80 °C until the following analysis. The individual liver samples were instead aliquoted and frozen at −80 °C waiting for subsequent investigation.

The experimental design involved the enzyme-linked immunosorbent assay (ELISA) on all the individual serum samples with the aim of discriminating between negative animals and animals who had suffered viremia. At this point, it was possible to identify two groups of individuals, positive and negative in the ELISA test, numerically balanced, and coming from the same farm, therefore comprising animals bred in the same environment with the same protocol in terms of housing and feeding. It is also important to underline the fact that the individuals involved in the positive group were selected not only on the basis of the positivity of the ELISA test described below but also by taking into consideration the specific content of HEV-IgG; this measure was necessary in order to involve in the study animals whose clinical course timing was comparable.

This preliminary investigation therefore made it possible to identify the two groups of animals to be subjected to comparative quantitative proteomics, exploiting for this analysis the liver tissue collected at the same time as the blood one.

### 2.2. Enzyme-Linked Immunosorbent Assay (ELISA) for the Identification of HEV Antibodies in Blood Serum

The HEV seroprevalence in pigs was evaluated by using an ELISA kit (PrioCHECK Porcine HEV Ab Strip kit; Applied Biosystems, Thermo Fisher Scientific, Inc., Foster City, CA, USA) specifically developed for the detection of HEV-IgG in blood serum. Samples (n = 136) were analyzed in triplicate following the manufacturer’s instructions. After the addition of the chromogen substrate in the test plates, the signal was read at 450 nm with an ELISA microplate reader (EnSpire 2300 multireader; PerkinElmer, Waltham, MA, USA).

For the results’ interpretation, reference was made to a cut-off value calculated by incorporating in the assay a positive control and a negative control. Values obtained above or equal to the cut-off were considered positive, and values below the cut-off threshold were considered negative, with an intermediate range in which the result must be considered doubtful and therefore requires further investigation.

### 2.3. Liver Exudate Collection and Filter-Aided Sample Preparation (FASP)

Liver exudate was obtained following the protocol previously used for other tissues [17]. Briefly, three cubes of about 10 g each were taken from the liver sampled for each animal. At this point, the samples thus prepared were centrifuged in 50 mL plastic tubes at 4 °C for 60 min at 4000× *g* (Mega Star 3.0, VWR International Srl, IT, Milan, Italy). After centrifugation, the exudate was collected in clean tubes analyzed in terms of total protein concentration, exploiting the Bradford colorimetric method and using BSA as a standard [18].

For each exudate sample, volumes corresponding to 100 µg of total proteins were taken and subjected to the filter-aided sample preparation (FASP) method [19,20]. Briefly, protein samples were diluted in 100 μL of denaturing buffer pH 8.5 composed of 7M urea, 2M thiourea, 30 Mm Tris and 4% CHAPS (Affymetrix/Thermo Fisher Scientific, Waltham, MA, USA). At this point, in order to promote reduction and alkylation in the exudate samples, dithiothreitol and iodoacetamide (Sigma-Aldrich/Merck, Saint Louis, MO, USA) were, respectively, added. Then, protein digestion was performed by using trypsin according to the FASP method [19,20]. The resulting peptides were finally purified by using C18 spin columns (Thermo Fisher Scientific, Waltham, MA, USA), dried under vacuum, and resuspended in 2% acetonitrile and 0.1% trifluoroacetic acid in order to proceed to the mass spectrometry.

### 2.4. Liquid Chromatography–Mass Spectrometry (LC-MS/MS) and Label-Free Quantitative Profiling

An Ultimate 3000 nanoRSLC system (Thermo Scientific, Waltham, MA, USA) coupled with an Orbitrap Fusion Tribrid™ mass spectrometer (Thermo Scientific, Waltham, MA, USA) was exploited in order to perform the LC–MS/MS evaluation. For the analysis, 1 µL of digest was loaded in a C18 trap column (C18 PepMap100; Thermo Scientific, Waltham, MA, USA) with subsequent desalting for 3 min and a flow rate equal to 25 μL/min in 0.1% (*v*/*v*) trifluoroacetic acid (TFA) and 2% (*v*/*v*) acetonitrile (ACN). Peptides were then flowed and resolved into the analytical column (Acclaim PepMap 100; Thermo Scientific, Waltham, MA, USA) by using a mobile phase made up of two solvents: A (0.1% (*v*/*v*) formic acid (FA)) and B (80% (*v*/*v*) ACN, 0.08% (*v*/*v*) FA). The applied gradient initially envisaged the use of 98% of A and 2% of B; subsequently, the percentage of B was increased up to 32% in a time interval of 75 min, and then further increased up to 90% in 5 min; this condition then remained stable for a further 5 min with a flow rate of 300 nL/min. The acquisition of MS1 and MS2 spectra was performed as recently described by Di Luca et al. [17].

The identification of proteins was performed by using the Proteome Discoverer (version 2.2; Thermo Fisher Scientific, Waltham, MA, USA) with the SEQUEST HT algorithm coupled with Percolator validation. MS files were searched against the UniProtKB-SwissProt Sus Scrofa database (http://www.uniprot.org/uniprot/, accessed on 27 November 2023). The search parameters were set as follows: peptide mass tolerance of 10 ppm; MS/MS mass tolerance of 0.6 Da; up to two missed cleavages were allowed; cysteine carbamidomethylation was set as a fixed modification; methionine oxidation was set as a variable modification. Only highly confident peptide identifications with a false discovery rate (FDR) ≤ 0.01 were considered.

Quantitative label-free analysis was carried out using version 2.0 of the software Progenesis QI for Proteomics (NonLinear Dynamics, London, UK). As previously described [21], the software exploits MS1 data in order to obtain quantitative information, and this is made by aligning the data based on the LC retention time of each sample to a reference file. This allows for drifts in retention times, giving an adjusted retention time for all runs in the analysis. The results were then filtered on the basis of the statistical analysis, and the software quantification algorithm calculated peptide abundance as the sum of the peak areas within its isotope boundaries. Each abundance value was then normalized (only peptide ions with charge states +1, +2 and +3 were allowed), permitting the calculation of the protein abundance as the sum of abundances of all peptide ions which had been identified as coming from the same protein. Peptides with a one-way ANOVA *p* value lower than ≤0.05 between experimental groups were exported and identified using Proteome Discoverer as described. Protein identifications were imported into Progenesis QI for proteomics and considered differentially expressed only complying the following criteria: proteins with a number of matched peptides higher or qual to 2, a fold chenge in abundance higher or equal to 1.5, and an ANOVA *p* value lower than 0.05 between the experimental groups.

### 2.5. Bioinformatics for Functional and Protein Network Analyses

The functional classification of proteins identified in both groups was performed by using the PANTHER (Protein ANalysis THrough Evolutionary Relationships) database system, released version 18.0 (http://www.pantherdb.org, accessed on 15 June 2023) [22]. The analysis for protein classification with reference to the biological processes was performed using default parameters and the annotations of *Sus Scrofa* genome as the background.

An enrichment analysis for KEGG (Kyoto Encyclopedia of Genes and Genomes) pathways and biological processes was instead carried out in order to obtain a functional interpretation of the differentially abundant proteins that were identified. Specifically, reference was made to SRplot (http://www.bioinformatics.com.cn, accessed on 15 June 2023), a web server for data analysis and visualization.

Proteins involved in selected pathways were then subjected to an in silico protein–protein interaction (PPI) analysis by exploiting the Search Tool for the Retrieval of Interacting Genes/Proteins (STRING) database (version 12.0; https://string-db.org, accessed on 29 June 2023) [23]. For the evaluation, reference was made to the Sus Scrofa specific interactome. Regarding the analytical parameters, the interaction score was set at 0.900, the highest confidence value permitted by the software to avoid false positives; furthermore, a high false discovery rate (FDR) stringency (1%) was selected.

### 2.6. Western Blot Analysis

Samples of liver exudate containing 25 µg of total proteins were mixed with a reducing sample buffer, and proteins were resolved by exploiting a 12% SDS-PAGE. Separated proteins were then trans-blotted onto polyvinylidene difluoride (PVDF) transfer membranes. The non-specific protein binding site on membranes was blocked by an overnight incubation at 4 °C in a solution containing 5% non-fat dry milk (Biorad, Milan, Italy) solubilized in TBS containing 0.2% Tween 20 (TBS-T). Subsequently, the PVDF was incubated at room temperature for 1 h with a monoclonal anti-Aldolase A primary antibody (sc-390733; Santa Cruz Biotechnology, Santa Cruz, CA, USA), 1:1000 diluted in a solution containing 1% non-fat dry milk solubilized in TBS-T. Membranes were then washed for 30 min in TBS-T (3 washes of 10 min each) and then incubated for 1 h at room temperature with a mouse IgG2 binding protein conjugated to horseradish peroxidase (sc-542731; Santa Cruz Biotechnology) diluted 1:10,000 in TBS-T containing 1% non-fat dry milk. After 30 min washing with TBS-T (3 washes of 10 min each), the immunoreactive bands were detected by inducing a chemiluminescence reaction (Westar ŋC Ultra 2.0; Cyanagen, IT, Bologna, Italy); then, images were acquired (Azure Biosystems C400, Dublin, CA, USA) and analyzed using the Image J software (1.54h) [24]. For each sample, the average band intensity corresponding to ALDOA was normalized to the average value obtained from the whole lane following a preliminary PVDF staining with Ponceau red; this step aimed to minimize any variations due to loading inaccuracies. Statistical analysis of the average band intensity of ALDOA was carried out across the two conditions (NG vs. PG) using ANOVA and Tukey’s test.

## 3. Results

### 3.1. Selection of Animals That Suffered the HEV Infection

An ELISA was used to estimate the seroprevalence of prior HEV infection in 136 pigs that were sampled at slaughter in the Abruzzo region (Italy) in the period of time between April 2022 and June 2022. The analysis allowed the identification of 99 positive animals (corresponding to 72.8% of the total) and 37 negative animals (27.2%), and no animals were classified as doubtful.

The data analysis also allowed the identification of 10 animals, 5 positive (positive group; PG) to the immunorecognition assay and 5 negative (negative group; NG), coming from the same farm. It is also important to reiterate that the individuals involved in the positive group were selected not only on the basis of the positivity of the ELISA test but even considering the specific amount of HEV-IgG; this measure was necessary in order to involve in the study animals whose clinical course timing was comparable.

This condition created the basis for the next phase described by the experimental design, in which a quantitative proteomics comparison was performed between two groups of animals homogeneous in terms of age, weight, breed, environment of origin, and breeding protocol (housing and feeding). Therefore, this constituted a context in which only the contact with HEV should represent the discriminating factor.

### 3.2. Protein Characterization in Pig Liver

**Figure 1:**
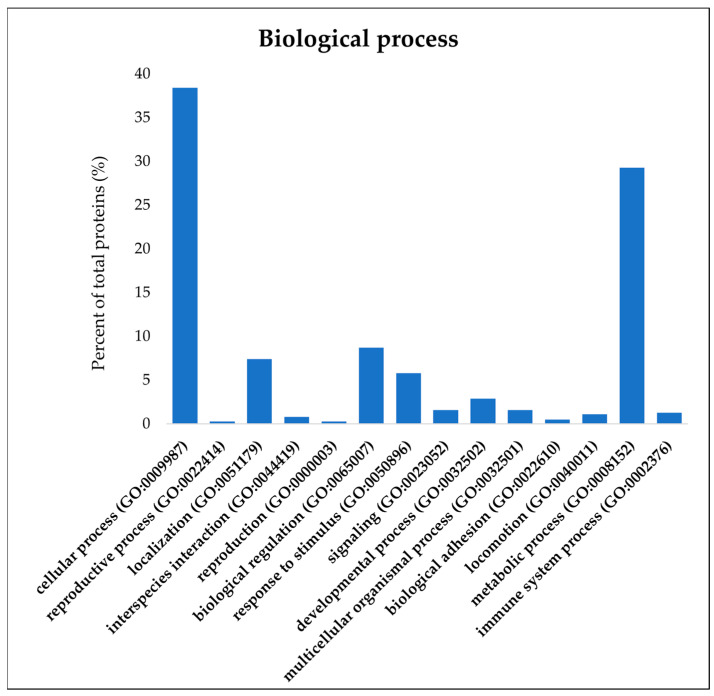
PANTHER (Protein ANalysis THrough Evolutionary Relationships) analysis of 1535 proteins discovered in all samples of pig liver exudate. The evaluation for protein classification with reference to the biological processes or gene ontology (GO) was performed using default parameters and the annotations of *Sus Scrofa* genome as the background.

Overall, the analysis of the proteome in all samples of pig liver exudate led to the identification of 4703 peptides belonging to 1535 proteins. The PANTHER analysis (Figure 1) made it possible to categorize these proteins based on their biological function, highlighting a majority of factors involved in the cellular process (38.4%), followed by proteins involved in the metabolic process (29.3%), biological regulation (8.7%), localization (7.4%), response to stimulus (5.8%), and other functions amounting to a percentage lower than 3%.

### 3.3. Comparative Proteomic Analysis of Pig Liver Exudate Negative and Positive to HEV Infection

The software incorporated in Progenesis QI for proteomics was used in order to investigate the differences at the proteome level between the group of pigs that suffered the HEV infection (PG) and subjects that tested negative to the ELISA evaluation (NG). The ranking of the proteins was performed by imposing specific conditions: the *p* value obtained by the one-way ANOVA (*p* < 0.05), fold change (≥1.5), and the number of matching peptides for each protein (>2).

Label-free data analysis was effective in identifying 554 proteins differentially expressed between the two experimental groups. In Table 1, the proteins with greater abundance in the PG samples are listed, while in Table 2, the proteins more represented in the NG exudates are reported. Given the large number of reported proteins, only those with a number of matching peptides greater than or equal to 10 are indicated in the aforementioned tables. The complete set of all proteins is reported in Supplementary Table S1 in the case of the 293 proteins with greater abundance in the PG samples, and Supplementary Table S2 in the case of the 261 most represented in the NG samples.

**Figure 2:**
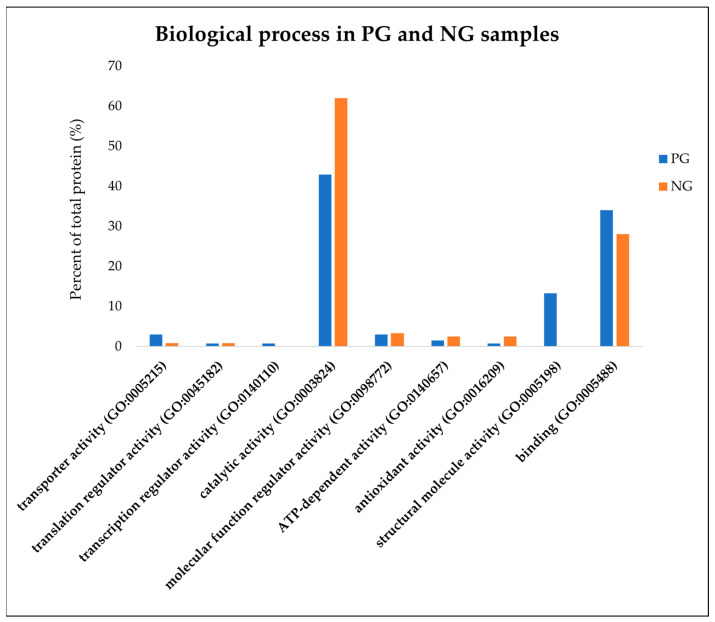
PANTHER (Protein ANalysis THrough Evolutionary Relationships) analysis on the subgroups of proteins discovered to be highly abundant in pig liver exudate obtained from the positive group (PG; blue bars) and the negative group (NG; orange bars). The evaluation for protein classification with reference to the biological processes or gene ontology (GO) was performed using default parameters and the annotations of *Sus Scrofa* genome as the background.

As reported in Figure 2, the PANTHER analysis was replicated on these subgroups of proteins in an attempt to perform a preliminary comparison concerning the biological process involved.

The main differences were observed for proteins involved in catalytic activity, with a greater abundance in the NG samples (43% vs. 62% in PG and NG, respectively), and in proteins involved in binding functions that were more represented in animals that suffered the infection (34.1% vs. 28.1% in PG and NG, respectively). Furthermore, it is very interesting that among the proteins over-expressed in the PG samples, there were factors associated with structural functions (13.3%), while among the proteins that were most abundant in the NG samples, these elements were totally missing.

### 3.4. Pathway Enrichment Analysis

An evaluation of the enriched pathways following the HEV infection was carried out in order to obtain a functional interpretation of the differentially abundant proteins that were identified. The analysis was performed by using SRplot, a web server for data analysis and visualization. Since the condition of interest on which to evaluate this parameter consists of the encounter between the host and HEV, the evaluation was conducted by associating each differentially expressed protein with its own fold change, which was entered into the software with a positive value in the case of more abundant proteins in the PG samples and with a negative value in the presence of the opposite condition.

**Figure 3:**
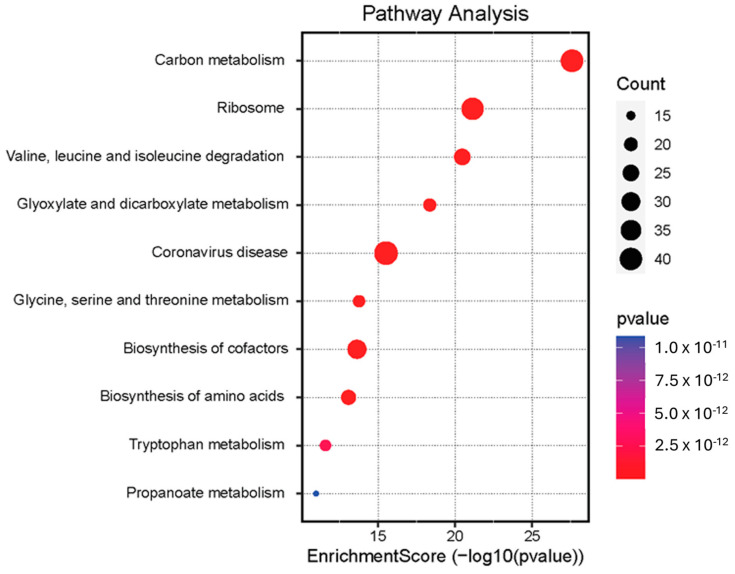
Pathway enrichment analysis performed on differentially expressed proteins by using the web server SRplot. The degree of enrichment of a pathway is associated with a score. Pathways with a high degree of enrichment are graphically represented with a red dot, which progresses to blue as the degree of enrichment decreases. The size of the dot is instead a function of the count of proteins involved in the corresponding pathway.

The analysis allowed us to highlight the effect of the interaction between HEV and the host biological system in inducing the potential enrichment of 69 pathways (the full list is reported in Supplementary Table S3). Figure 3 shows a graphic representation of the top 10 enriched pathways resulting from the analysis. Among these, carbon metabolism stands out, followed by ribosomal activity, degradation of valine, leucine, and isoleucine, glyoxylate and dicarboxylate metabolism, and, interestingly, the pathway related to coronavirus disease, therefore constituting a sign of the stimulation of pathways associated with a viremia response.

As highlighted in Figure 3, the pathways relating to carbon metabolism, ribosomal activity, and mechanisms associated with coronavirus disease are also those in which a greater number of proteins are involved among those that are differentially expressed and identified with proteomic investigation. Table 3 shows the exact indication of the proteins involved in the cited pathways.

### 3.5. Evaluation of Protein–Protein Interaction (PPI)

**Figure 4:**
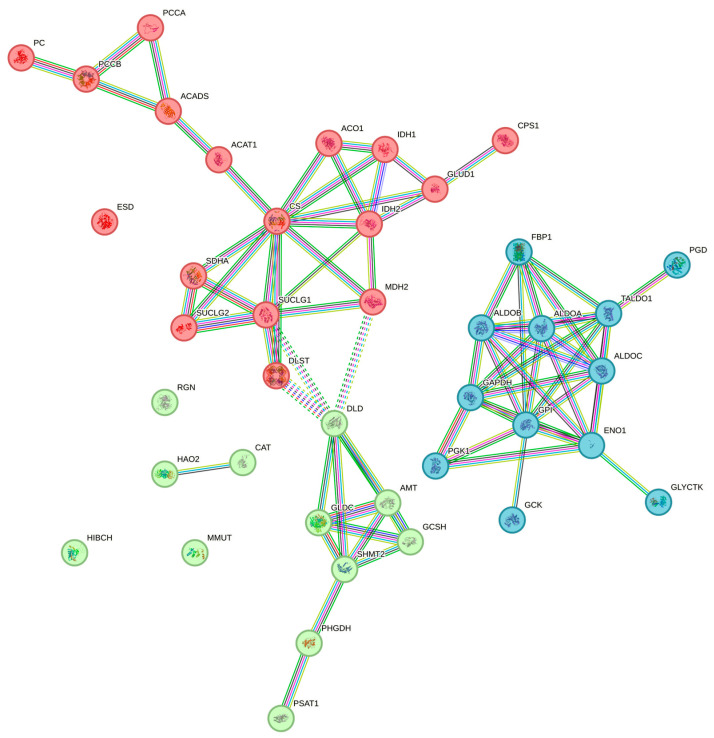
Protein–protein interaction analysis of proteins involved in the enrichment of the pathway related to carbon metabolism. The evaluation was performed by using STRING and involved 41 proteins. Interactions are shown in different colors: cyan is from curated databases, magenta is experimentally determined, dark green is gene neighborhood, red is gene fusion, blue is gene co-occurrence, light green is text mining, black is coexpression, and light blue is protein homology.

The analysis with STRING represented a useful tool to better characterize the data regarding the enrichment of the pathways. Attention was specifically focused on carbon metabolism, highlighting the potential interactions between the differentially expressed proteins that have been indicated to contribute to this biological mechanism. Figure 4 shows the interactome between the proteins that were assigned to the carbon metabolism pathway via SRplot.

The graphic evaluation of the interactions shown allowed us to identify the most important factors associated with the reference pathway. In particular, glucose-6-phosphate isomerase (GPI) seems to have the possibility of performing nine interactions, followed by mitochondrial citrate synthase (CS) with eight interactions, and glyceraldehyde-3-phosphate dehydrogenase (GAPDH) and fructose-bisphosphate aldolase A (ALDOA) with seven.

### 3.6. Confirmation of the Differential Expression of Fructose-Bisphosphate Aldolase a by Western Blot

**Figure 5:**
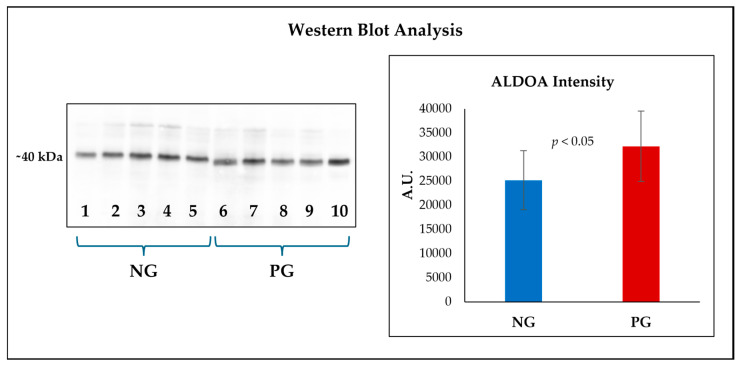
Representative Western blot analysis of fructose-bisphosphate aldolase A (ALDOA) in liver exudate obtained from pigs seronegative (NG; blue bars) and seropositive (PG; red bars) for HEV. The membranes show an increment in the band intensity, expressed as arbitrary units (A.U.), in PG samples (*p* < 0.05).

In light of the results obtained from quantitative proteomic and bioinformatic analyses, we chose to confirm the differential expression of ALDOA by an immunorecognition approach. The Western blot (Figure 5) in fact highlighted a greater presence of the enzyme (*p* < 0.05) in the samples obtained from animals that had come into contact with HEV.

## 4. Discussion

The present study addresses the identification of changes induced by prior HEV infection on the porcine liver proteome. The intent was therefore to first obtain useful information to better understand the biochemical mechanisms deriving from this event, as well as identify potential protein markers useful for discriminating between healthy animals and animals positive for the virus or which, in any case, have suffered from viremia. Specifically, we focused our attention on the Abruzzo region, a geographical area located in Central Italy, which was reported to be characterized by a high seroprevalence (about 49%) of HEV among human blood donors [25]. The prevalence of HEV infection in this specific geographical area was the subject of a detailed evaluation performed by Picchi et al. [26]. Briefly, the authors showed that more than one third of acute non-ABC hepatitis cases are caused by HEV and that all cases are autochthonous, as proven by the HEV-3 genotype. Specifically, genotype 3 and its subtypes appear to have wide circulation in foods, which act as preferential carriers of the virus and favor potential transmission in humans among countries. However, the same authors, in the study period, did not find significant differences between populations regarding work, comorbidities, and additional risk factors such as contact with food or animals. Specifically, in the patients being evaluated, employment in livestock farming was rare, while aspects related to animal contact, gardening, as well as the consumption of pork sausages and, specifically, pork liver sausages, represented widespread habits without intra-regional differences. Precisely, the wide diffusion of these critical elements has been indicated as the main reason that could explain the lack of significance of these risk factors in the area of reference, although it could be useful in a comparison investigation with other regions in which there is a different HEV seroprevalence.

Within the 136 animals involved, the ELISA approach identified 99 individuals with anti-HEV antibodies. The prevalence therefore stood at around 73%, a percentage nevertheless lower if compared with what was reported in the study performed by Martino et al. [27], who, in the same geographical area of reference, calculated an overall seroprevalence of 93.9% among 233 pig serum samples collected in different slaughterhouses.

The pig liver represents a matrix that, over time, has been the subject of several proteomic studies. However, with specific reference to HEV, it must be said that there are not many studies, and some of these refer to gel-based proteomic approaches in which, after 2D analysis, protein spots were enzymatically digested in-gel and subsequently analyzed [12,28]. In our investigation, the application of the LC-MS/MS profiling allowed the selection of 554 proteins differentially expressed between the two experimental groups: 293 proteins with greater abundance in PG samples and 261 more represented in NG exudates. This is a notable difference that testifies to the fact that differences among animals that have interacted or not with HEV may involve different aspects and functions of the entire proteome, with variations that presumably represent the result of a reprogramming of the genome following the interaction with the virus. This aspect falls within the scope of phenotypic plasticity, which represents the mechanism by which organisms should be able to survive in the face of unpredictable environmental stress. With specific reference to viruses, they can induce changes in the host’s physiological homeostasis, hence allowing a better adaptation to different ecological niches for the organisms involved [29,30].

When analyzing the function of the differentially expressed proteins, it emerged that the proteins over-expressed in the PG samples were more associated with transporter activity, binding, and structural functions, while in the NG samples, factors mainly involved in catalytic activity prevailed. Deciphering the meaning of these variations observed in functional groups of proteins is extremely complex, and this is the reason for which a complementary analysis of pathway enrichment was performed. This approach made it possible to highlight the effect of the interaction between HEV and the host biological system in inducing the potential enrichment of 69 pathways, among which carbon metabolism, ribosomal activity, and, interestingly, the response to coronavirus disease stand out.

The finding regarding the enrichment of carbon metabolism involves a total of 41 proteins, which were analyzed in order to evaluate their mutual interactions. This approach allowed us to focus attention on four specific elements that appear to have a central role in this pathway: glucose-6-phosphate isomerase (GPI), mitochondrial citrate synthase (CS), glyceraldehyde-3-phosphate dehydrogenase (GAPDH), and fructose-bisphosphate aldolase A (ALDOA). GPI, GAPDH, and ALDOA are all enzymes involved in the glycolytic breakdown of glucose. Specifically, GPI catalyzes the conversion of glucose-6-phosphate to fructose-6-phosphate in the cytoplasm, the second step in glycolysis, and the reverse reaction during gluconeogenesis. ALDOA is a ubiquitous cytosolic enzyme that catalyzes the fourth step of glycolysis, in which there is the conversion of fructose 1,6-bisphosphate in glyceraldehyde-3-phosphate and dihydroxyacetone phosphate; as for the previous enzyme, ALDOA also performs the reverse function in gluconeogenesis. GAPDH is instead an enzyme of about 37 kDa that is responsible for the sixth step of glycolysis that provides for the conversion of glyceraldehyde 3-phosphate to D-glycerate 1,3-bisphosphate [31]. Overall, it is therefore quite clear that the interaction with HEV induces a strengthening of the process which involves the breakdown of glucose in order to obtain energy and carbon residues useful for virus survival. This aspect has been already addressed by Moin et al. [32] who hypothesized a pivotal role of the viral Open Reading Frame 3 (ORF3), which encodes a protein that was reported to be associated with the cytoskeleton through a hydrophobic N-terminal region [33]. Its main function appears to be associated with promoting the survival of HEV following infection; however, through studies conducted on Huh7 cells, ORF3 was shown not to be required for viral replication, thus suggesting an accessory role primarily useful in the regulation of the host response to infection [34]. Moin et al. [32] specifically performed experiments of protein profiling on cultured cells modified to constitutively express the ORF3 proteins. This condition allowed an observation of the differential expression of various metabolic pathway proteins, including several glycolytic enzymes. In particular, the expression of the genes coding for these enzymes appeared to be induced by the binding of hypoxia-inducible factor (HIF) to an HIF-responsive element (HRE) at the level of the genes’ promoter regions. In reference to the potential enhancement of glucose metabolism observed in the liver tissue, it is, however, necessary to add considerations also associated with the possible effects on pork quality. From this point of view, it is well known and characterized that immediately after the animal is killed, the pig muscle is converted into meat through the biochemical process of glycolysis. This mechanism involves, specifically, the accumulation of lactate with a consequent decline in the pH value at the tissue level. This variation is closely related to the denaturation of actin and myosin in the muscle, with a consequent alteration of the muscle protein matrix which finds expression with even quite marked variations in meat color, toughness, and water-holding capacity [35]. For this reason, glycolytic potential is considered a reliable and rapid indicator of meat quality [36], and the fact that HEV infection has enhanced this pathway in the liver makes it plausible that the same mechanism can occur in other tissues, including muscle. This would lead to a qualitative impoverishment of animal production, therefore representing a further critical aspect linked to the circulation of the virus in livestock farming.

All this is therefore part of the strategy commonly applied by viruses to survive through the modulation of the host signaling pathways. Viruses in fact possess a limited protein repertoire, which makes it necessary for them to exploit host biochemical functions. From this perspective, the need to understand the data regarding the enrichment associated with ribosomal function highlighted by the bioinformatic analysis is therefore quite immediate. The viremic process is generally related to the exploitation of the cellular ribosomal apparatus, and this aspect is so characterized and consolidated that, in the last decade, different anti-viral strategies aimed at controlling this mechanism have been developed; among them, the introduction of ribosome-inactivating proteins (RIPs), i.e., rRNA N-glycosylases obtained from plants with the ability to inactivate ribosomes, thus inhibits the synthesis of proteins useful for the microorganism [37].

A further aspect of interest that emerged from the bioinformatic analysis of the data obtained from the comparative proteomics investigation concerned the enrichment of the response pathway to coronavirus infection. This obviously represents a singular element that deserves further investigation, although it should be noted that these two viruses present some important homologies. First, a coronavirus is characterized by sequences with high similarity to the HEV ORF1; this sequence should code for an ADP ribose-1’ phosphatase responsible for the removal of the phosphate group of ADPr-1-phosphate in the tRNA-splicing pathway. In addition to this, in HEV, there is a helicase belonging to the SF-1 family, which is also found in other positive-strand RNA viruses including coronaviruses [38]. Based on the results of pathway enrichment analysis, it is clear that, even for this pathway, there is a large involvement of factors associated with ribosomal functionality, with the addition of fibrinogen, which is downregulated following viral action. Fibrinogen represents a hepatic acute-phase protein that serves as a central factor in the maintenance of host homeostasis during an acute-phase response. In this context, for a better understanding of the observed finding, one could refer to the study performed by Ratra et al. [39], who highlighted the ability of HEV ORF3 to interact with fibrinogen in mammalian cells. The establishment of this interaction therefore seems to be effective in inducing a lowering of fibrinogen levels, which seems to be justified by a transcriptional downregulation of the levels of the mRNA corresponding to the constituent chains. Another very interesting aspect concerns the fact that in the presence of HEV, the expression of fibrinogen is insensitive to interleukin 6 (IL-6), which normally represents a positive mediator of fibrinogen during an acute-phase response.

In light of the results obtained from quantitative proteomic and bioinformatic analyses, we chose to confirm the differential expression of ALDOA by exploiting Western blot analyses. The specific choice to focus attention on this enzyme derives first of all from its prominent role in the glycolytic process, as well as from the role played in influencing other biochemical mechanisms, one of which is the modulation of transcriptional mechanisms [40]. Overall, we believe that this protein may represent a common thread between the two pathways found to be most enriched in pigs following contact with HEV, namely carbon metabolism and ribosomal activity. We know that the transcription process in eukaryotic systems is mediated by three RNA polymerases. Among these, RNA polymerase III (Pol III) was reported to be involved in the synthesis of tRNA and other untranslated RNAs [41]. Cieśla et al. [40] conducted a study in which it was found that ALDOA overproduction induced a missense mutation in a gene encoding for the second largest subunit of the Pol III complex. This event appears to be correlated with the enhanced de novo transcription of tRNA, a condition which therefore should allow an acceleration of ribosomal function. Also very interesting was the data regarding the fact that the overproduction of ALDOA in an inactive form still induced the same phenomenon, indicating that the influence of this glycolytic enzyme on Pol III transcription is independent from its catalytic function. The Western blot analysis effectively confirmed the greater presence of ALDOA in the PG samples, giving support to the bioinformatic findings regarding the enrichment of the pathways associated with carbon metabolism and ribosomal activity, with good confidence that these events may be interconnected with each other.

Overall, the application of quantitative proteomics proved effective in highlighting elements that could characterize the interaction between HEV and porcine metabolism. Above all, it seems quite clear how the virus has stimulated processes useful for its survival, such as the breakdown of glucose to obtain energy and carbon residues and the ribosomal function useful for protein synthesis. In conclusion, it is therefore believed that the label-free LC-MS/MS approach made it possible to obtain crucial information in identifying a viral signature on the host metabolism.

## Figures and Tables

**Table 1:** Proteins identified with greater abundance in samples obtained from pigs that suffered the HEV infection (PG) following label-free MS/MS analysis (Progenesis QI for proteomics).

UniProt ID ^1^	Gene Name	Protein Description	Peptide ^2^	Score ^3^	Anova (*p*)	Fold Change
A0A481B0J7	CPS1	carbamoyl-phosphate synthase [ammonia], mitochondrial	78	457.27	0.0049	2.04
A0A8D1CLI4	FLNA	filamin-A isoform X2	27	99.35	0.0010	2.12
A0A480SKT5	DMGDH	dimethylglycine dehydrogenase, mitochondrial isoform X1	25	125.00	0.0061	2.75
A0A287ATN8	HSPD1	60 kDa heat shock protein, mitochondrial	24	147.20	0.0039	1.98
P42174	GLUD1	glutamate dehydrogenase 1, mitochondrial	23	133.50	0.0007	2.01
Q7YS28	PC	pyruvate carboxylase, mitochondrial	23	113.27	0.0033	10.54
A0A4X1URZ5	ACSS3	acyl-CoA synthetase medium-chain family member 4	22	121.18	0.0017	2.01
A0A480SA59	SARDH	sarcosine dehydrogenase, mitochondrial isoform X1	22	107.97	0.0004	2.96
A0A287ADJ2	HSPA9	stress-70 protein, mitochondrial	20	84.28	0.0001	2.50
Q2XQV4	ALDH2	aldehyde dehydrogenase, mitochondrial precursor	18	94.69	0.0026	1.78
A0A4X1UM84	PCK1	phosphoenolpyruvate carboxykinase, cytosolic [GTP]	18	98.33	0.0036	2.90
P26234	VCL	Vinculin	18	63.18	0.0007	2.28
P0DTA4	PCCA	propionyl-CoA carboxylase alpha chain, mitochondrial	18	78.68	0.0025	1.98
A0A8D1MNH9	SPTAN1	spectrin alpha chain, non-erythrocytic 1	17	65.06	0.0000	2.68
P33198	IDH2	isocitrate dehydrogenase, mitochondrial	16	66.34	0.0113	1.53
P79384	PCCB	propionyl-CoA carboxylase beta chain, mitochondrial precursor	16	70.50	0.0173	1.63
A0A8D1EFW2	AGXT2	alanine-glyoxylate aminotransferase 2, mitochondrial	16	87.66	0.0012	1.98
D0G0B3	ACAA2	3-ketoacyl-CoA thiolase, mitochondrial	16	99.47	0.0057	2.21
A0A4X1UM84	PCK1	phosphoenolpyruvate carboxykinase, cytosolic [GTP]	16	94.81	0.0055	1.90
A0A287BBX3	NADK2	NAD kinase 2, mitochondrial	16	76.07	0.0016	1.98
O19072	OTC	ornithine transcarbamylase, mitochondrial	15	94.18	0.0012	2.04
P50441	GATM	glycine amidinotransferase, mitochondrial	15	92.88	0.0003	2.22
A0A4X1V4W9	HSDL2	hydroxysteroid dehydrogenase-like protein 2	15	68.16	0.0003	2.69
I3LJ48	EHHADH	peroxisomal bifunctional enzyme	14	61.42	0.0007	2.01
A0A8D1GE69	ACADSB	short/branched chain specific acyl-CoA dehydrogenase, mitochondrial	14	80.79	0.0102	2.04
A0A8D0XUW7	ATP5F1B	ATP synthase subunit beta, mitochondrial	14	65.54	0.0032	3.05
A0A287AIP7	ACSL1	long-chain-fatty-acid--CoA ligase 1	13	48.59	0.0061	1.73
A0A4X1URZ5	ACSS3	acyl-CoA synthetase short-chain family member 3, mitochondrial	13	55.80	0.0003	3.38
P80229	SERPINB1	leukocyte elastase inhibitor	12	48.80	0.0026	3.98
A0A287BLE0	FLNB	filamin-B	12	44.89	0.0023	2.03
C0MHR2	CLTC	clathrin heavy chain	12	45.85	0.0218	2.28
A0A8D1UET6	TST	thiosulfate sulfurtransferase	12	75.14	0.0022	1.89
A0A286ZND5	PRDX1	peroxiredoxin-1	12	60.03	0.0087	2.88
P80021	ATP5F1A	ATP synthase subunit alpha, mitochondrial	12	65.71	0.0066	2.73
Q29554	HADHA	trifunctional enzyme subunit alpha, mitochondrial	11	44.65	0.0039	4.12
F1RMF7	GLYAT	glycine N-acyltransferase	11	56.90	0.0150	1.58
I3LP02	ACAT1	acetyl-CoA acetyltransferase, mitochondrial	11	54.70	0.0119	1.87
Q6UAQ8	ETFB	electron transfer flavoprotein subunit beta	11	54.83	0.0067	2.04
Q9XT00	HSD17B8	3-hydroxyacyl-CoA dehydrogenase	11	85.09	0.0000	2.42
F2Z5N5	RPL26L1	ceruloplasmin isoform X1	10	59.12	0.0027	2.46
F1SAD9	PDIA4	protein disulfide-isomerase A4	10	36.38	0.0269	2.21
P53590	SUCLG2	succinate-CoA ligase [GDP-forming] subunit beta, mitochondrial	10	58.96	0.0036	2.32
B2ZF49	HADHA	enoyl-CoA hydratase, mitochondrial	10	48.23	0.0102	1.89
P00346	MDH2	malate dehydrogenase, mitochondrial	10	63.15	0.0006	1.89
A0A4X1UTH9	IVD	isovaleryl-CoA dehydrogenase, mitochondrial	10	62.96	0.0032	2.02
P41367	ACADM	medium-chain specific acyl-CoA dehydrogenase, mitochondrial	10	47.06	0.0003	2.85
A0A8W4F721	ALDH4A1	delta-1-pyrroline-5-carboxylate dehydrogenase, mitochondrial	10	42.48	0.0035	1.93

^1^ accession number in the UniProt database; ^2^ matching peptides for each protein, used for quantitation; ^3^ SEQUEST score. The presence of the notation “Infinity” in the fold change column indicates the presence of the protein only in the PG samples.

**Table 2:** Proteins identified with greater abundance in samples obtained from pigs that tested negative in the ELISA evaluation (NG) following label-free MS/MS analysis (Progenesis QI for proteomics).

UniProt ID ^1^	Gene Name	Protein Description	Peptide ^2^	Score ^3^	Anova (*p*)	Fold Change
Q58GK8	FASN	fatty acid synthase	24	124.48	0.0194	1.82
Q95332	BHMT	betaine-homocysteine S-methyltransferase 1	19	140.67	0.0003	1.78
L7TEV7	AOX1	aldehyde oxidase	18	91.88	0.0072	2.06
F1S1E7	DPYS	dihydropyrimidinase isoform X1	17	108.90	0.0015	1.86
A0A4X1VFQ7	LOC110255172	acyl-coenzyme A amino acid N-acyltransferase 2-like	17	93.37	0.0078	1.75
A0A480ST43	ASS1	argininosuccinate synthase	16	97.76	0.0015	1.66
F1S3H8	RDH8	retinol dehydrogenase 1	16	76.58	0.0276	1.54
A0A4X1UZ96	UPB1	beta-ureidopropionase	16	94.43	0.0086	1.98
P23687	PREP	prolyl endopeptidase	16	71.89	0.0139	2.11
Q28943	DPYD	dihydropyrimidine dehydrogenase [NADP(+)] precursor	15	67.95	0.0017	1.98
Q06AA3	RGN	regucalcin	14	94.49	0.0034	1.71
A0A5G2QAG3	LAP3	cytosol aminopeptidase isoform X1	14	79.04	0.0016	1.78
P46410	GLUL	glutamine synthetase isoform X1	13	70.53	0.0008	3.78
A0A480R4L3	KYNU	kynureninase isoform X1	13	50.36	0.0008	2.24
A0A286ZZN9	GLMS	glutamine-fructose-6-phosphate aminotransferase 1	13	58.72	0.0000	2.19
P37111	ACY1	aminoacylase-1	12	86.53	0.0009	2.34
Q6Q2C2	EPHX2	bifunctional epoxide hydrolase 2	12	54.89	0.0174	1.66
A0A286ZRS0	GSS	glutathione synthetase	11	44.56	0.0071	1.91
P28839	LAP3	xaa-Pro dipeptidase	11	48.18	0.0023	2.64
A0A4X1TBE8	GSTM3	glutathione S-transferase	11	49.20	0.0023	1.72
I3L804	YARS1	tyrosine-tRNA ligase, cytoplasmic isoform X1	11	41.54	0.0005	2.35
I3LIM2	UGDH	UDP-glucose 6-dehydrogenase	11	45.24	0.0098	1.82
Q02110	HPD	4-hydroxyphenylpyruvate dioxygenase	11	55.49	0.0033	1.65
D2SW95	COPB1	coatomer subunit beta	11	40.14	0.0082	1.67
A0A8D0YFM9	GSR	glutathione reductase, mitochondrial	10	58.65	0.0093	1.98
A0A480NMC5	DPP3	dipeptidyl peptidase 3 isoform X1	10	53.31	0.0009	2.71
O97788	FABP4	fatty acid-binding protein	10	54.41	0.0011	1.67
A0A4X1TZD5	EEF1A	elongation factor 1-alpha 1	10	61.46	0.0008	2.28
P03974	VCP	transitional endoplasmic reticulum ATPase	10	45.68	0.0014	2.04
F1SNJ5	AKR1D1	3-oxo-5-beta-steroid 4-dehydrogenase isoform X1	10	52.58	0.0026	1.75
F1SIJ9	PSAT1	phosphoserine aminotransferase	10	59.02	0.0008	1.63

^1^ accession number in the UniProt database; ^2^ matching peptides for each protein, used for quantitation; ^3^ SEQUEST score. The presence of the notation “Infinity” in the fold change column indicates the presence of the protein only in the NG samples.

**Table 3:** Pathway enrichment analysis performed on differentially expressed proteins by using the web server SRplot. The table specifically shows the evaluation relating to carbon metabolism, ribosomal activity, and mechanisms associated with coronavirus disease.

Enriched Pathway Description	*p* Value	*p* Adjust	q Value	GeneID	Protein Count
Carbon metabolism	2.60 × 10^−14^	6.16 × 10^−12^	4.60 × 10^−12^	CPS1, GLUD1, PC, PCCA, IDH2, PCCB, ACAT1, SUCLG2, MDH2, ACADS, CS, ACO1, DLD, FBP1, SHMT2, SUCLG1, GLDC, SDHA, MMUT, TALDO1, GLYCTK, AMT, DLST, HIBCH, HAO2, ALDOA, ALDOC, GCSH, RGN, PSAT1, PGK1, GPI, CAT, GAPDH, PHGDH, IDH1, ENO1, ESD, ALDOB, GCK, PGD	41
Ribosome	7.46 × 10^−8^	8.84 × 10^−6^	6.60 × 10^−7^	RPL26L1, RPS3, RPLP0, RPSA, RPL7, RPS4X, RPS3A, RPL9, RPL7A, RPL18, RPL30, RPS8, RPL4, RPS19, RPL13A, RPL19, RPL5, RPS7, RPS9, RPL10A, RPL27A, RPS18, RPS25, RPL24, RPS23, RPL8, RPS13, RPL37A, RPS21, RPL10L, RPL36, RPL35A, RPL23, RPS24, RPL6, RPS11, RPS17, RPS10, RPL22, RPS12	40
Coronavirus disease	3.13 × 10^−2^	1.49 × 10^−1^	1.11 × 10	RPL26L1, RPS3, RPLP0, RPSA, RPL7, RPS4X, RPS3A, RPL9, STAT1, RPL7A, RPL18, RPL30, RPS8, RPL4, RPS19, RPL13A, RPL19, RPL5, RPS7, RPS9, RPL10A, RPL27A, RPS18, RPS25, FGB, RPL24, RPS23, RPL8, RPS13, RPL37A, RPS21, RPL10L, RPL36, RPL35A, C8A, RPL23, RPS24, RPL6, RPS11, RPS17, RPS10, RPL22, RPS12, FGG	44

## Data Availability

Data are available after request to the corresponding author.

## Supplementary Materials

The following supporting information can be downloaded at: https://www.mdpi.com/article/10.3390/v16030408/s1, Table S1: Proteins identified with greater abundance in samples obtained from pigs that suffered the HEV infection (PG) following label-free MS/MS analysis (Progenesis QI for proteomics). Table S2: Proteins identified with greater abundance in samples obtained from pigs that resulted negative to the ELISA evaluation (NG) following label-free MS/MS analysis (Progenesis QI for proteomics). Table S3: Pathway enrichment analysis performed on differentially expressed proteins by using the web server SRplot.
