# Supplementary material for: Label-Free Quantitative Analysis of Pig Liver Proteome after Hepatitis E Virus Infection
Source: Viruses. 2024 Mar 6;16(3):408. doi: 10.3390/v16030408 (PMC10976091; doi:10.3390/v16030408)
Supplement: Supplementary file 1 [file viruses-16-00408-s001.zip › Supplementary_Table_S1_viruses-2770250.pdf]

Supplementary Table S1. Proteins identified with greater abundance in samples obtained from pigs that suffered the HEV infection (PG) following label-free MS/MS analysis (Progenesis QI for proteomics).

UniProt ID[1]	Gene name	Protein Description	Peptide[2]	Score[3]	Anova (p)	Fold change
A0A481B0J7	CPS1	carbamoyl-phosphate synthase [ammonia], mitochondrial	78	457.27	0.0049	2.04
A0A8D1CLI4	FLNA	filamin-A isoform X2	27	99.35	0.0010	2.12
A0A480SKT5	DMGDH	dimethylglycine dehydrogenase, mitochondrial isoform X1	25	125.00	0.0061	2.75
A0A287ATN8	HSPD1	60 kDa heat shock protein, mitochondrial	24	147.20	0.0039	1.98
P42174	GLUD1	glutamate dehydrogenase 1, mitochondrial	23	133.50	0.0007	2.01
Q7YS28	PC	pyruvate carboxylase, mitochondrial	23	113.27	0.0033	10.54
A0A4X1URZ5	ACSS3	acyl-CoA synthetase medium-chain family member 4	22	121.18	0.0017	2.01
A0A480SA59	SARDH	sarcosine dehydrogenase, mitochondrial isoform X1	22	107.97	0.0004	2.96
A0A287ADJ2	HSPA9	stress-70 protein, mitochondrial	20	84.28	0.0001	2.50
Q2XQV4	ALDH2	aldehyde dehydrogenase, mitochondrial precursor	18	94.69	0.0026	1.78
A0A4X1UM84	PCK1	phosphoenolpyruvate carboxykinase, cytosolic [GTP]	18	98.33	0.0036	2.90
P26234	VCL	Vinculin	18	63.18	0.0007	2.28
P0DTA4	PCCA	propionyl-CoA carboxylase alpha chain, mitochondrial	18	78.68	0.0025	1.98
A0A8D1MNH9	SPTAN1	spectrin alpha chain, non-erythrocytic 1	17	65.06	0.0000	2.68
P33198	IDH2	isocitrate dehydrogenase, mitochondrial	16	66.34	0.0113	1.53
P79384	PCCB	propionyl-CoA carboxylase beta chain, mitochondrial precursor	16	70.50	0.0173	1.63
A0A8D1EFW2	AGXT2	alanine-glyoxylate aminotransferase 2, mitochondrial	16	87.66	0.0012	1.98
D0G0B3	ACAA2	3-ketoacyl-CoA thiolase, mitochondrial	16	99.47	0.0057	2.21
A0A4X1UM84	PCK1	phosphoenolpyruvate carboxykinase, cytosolic [GTP]	16	94.81	0.0055	1.90
A0A287BBX3	NADK2	NAD kinase 2, mitochondrial	16	76.07	0.0016	1.98
O19072	OTC	ornithine transcarbamylase, mitochondrial	15	94.18	0.0012	2.04
P50441	GATM	glycine amidinotransferase, mitochondrial	15	92.88	0.0003	2.22
A0A4X1V4W9	HSDL2	hydroxysteroid dehydrogenase-like protein 2	15	68.16	0.0003	2.69
I3LJ48	EHHADH	peroxisomal bifunctional enzyme	14	61.42	0.0007	2.01
A0A8D1GE69	ACADSB	short/branched chain specific acyl-CoA dehydrogenase, mitochondrial	14	80.79	0.0102	2.04
A0A8D0XUW7	ATP5F1B	ATP synthase subunit beta, mitochondrial	14	65.54	0.0032	3.05
A0A287AIP7	ACSL1	long-chain-fatty-acid--CoA ligase 1	13	48.59	0.0061	1.73
A0A4X1URZ5	ACSS3	acyl-CoA synthetase short-chain family member 3, mitochondrial	13	55.80	0.0003	3.38
P80229	SERPINB1	leukocyte elastase inhibitor	12	48.80	0.0026	3.98
A0A287BLE0	FLNB	filamin-B	12	44.89	0.0023	2.03
C0MHR2	CLTC	clathrin heavy chain	12	45.85	0.0218	2.28
A0A8D1UET6	TST	thiosulfate sulfurtransferase	12	75.14	0.0022	1.89
A0A286ZND5	PRDX1	peroxiredoxin-1	12	60.03	0.0087	2.88

P80021	ATP5F1A	ATP synthase subunit alpha, mitochondrial	12	65.71	0.0066	2.73
Q29554	HADHA	trifunctional enzyme subunit alpha, mitochondrial	11	44.65	0.0039	4.12
F1RMF7	GLYAT	glycine N-acyltransferase	11	56.90	0.0150	1.58
I3LP02	ACAT1	acetyl-CoA acetyltransferase, mitochondrial	11	54.70	0.0119	1.87
Q6UAQ8	ETFB	electron transfer flavoprotein subunit beta	11	54.83	0.0067	2.04
Q9XT00	HSD17B8	3-hydroxyacyl-CoA dehydrogenase	11	85.09	0.0000	2.42
F2Z5N5	RPL26L1	ceruloplasmin isoform X1	10	59.12	0.0027	2.46
F1SAD9	PDIA4	protein disulfide-isomerase A4	10	36.38	0.0269	2.21
P53590	SUCLG2	succinate-CoA ligase [GDP-forming] subunit beta, mitochondrial	10	58.96	0.0036	2.32
B2ZF49	HADHA	enoyl-CoA hydratase, mitochondrial	10	48.23	0.0102	1.89
P00346	MDH2	malate dehydrogenase, mitochondrial	10	63.15	0.0006	1.89
A0A4X1UTH9	IVD	isovaleryl-CoA dehydrogenase, mitochondrial	10	62.96	0.0032	2.02
P41367	ACADM	medium-chain specific acyl-CoA dehydrogenase, mitochondrial	10	47.06	0.0003	2.85
A0A8W4F721	ALDH4A1	delta-1-pyrroline-5-carboxylate dehydrogenase, mitochondrial	10	42.48	0.0035	1.93
O02734	HMGCS2	hydroxymethylglutaryl-CoA synthase, mitochondrial	9	38.04	0.0030	12.50
A0A8D1Q766	ADH1	alcohol dehydrogenase 1	9	35.98	0.0025	2.76
A0A8D0Q9R1	MTTP	microsomal triglyceride transfer protein large subunit precursor	9	27.18	0.0078	2.40
A0A5G2QRE7	ETFA	electron transfer flavoprotein subunit alpha, mitochondrial	9	50.72	0.0116	2.01
Q0Z8U2	RPS3	40S ribosomal protein S3	8	27.35	0.0078	2.46
A0A8D1VUA0	HADHB	trifunctional enzyme subunit beta, mitochondrial	8	27.85	0.0065	4.76
A0A480XQL7	EIF4A2	eukaryotic initiation factor 4A-II	8	27.13	0.0140	1.76
P81140	GCDH	glutaryl-CoA dehydrogenase, mitochondrial	8	38.22	0.0094	2.69
O46409	APOA4	apolipoprotein A-IV precursor	8	24.58	0.0063	24.87
A0A5G2QNM8	COPG1	coatomer subunit gamma-1	8	25.80	0.0283	2.72
Q6XGY2	DECR	2,4-dienoyl-CoA reductase, mitochondrial	8	34.16	0.0056	1.72
P39092	A1BG	alpha-1B-glycoprotein	8	35.00	0.0049	2.11
F6QA08	PDIA3	protein disulfide-isomerase A3	8	28.00	0.0270	1.57
A0A480EI27	AK2	adenylate kinase 2, mitochondrial isoform X1	8	35.00	0.0001	2.92
F1SA74	MTHFD1	C-1-tetrahydrofolate synthase, cytoplasmic	8	35.51	0.0015	1.71
P79273	ACADS	short-chain specific acyl-CoA dehydrogenase, mitochondrial	8	38.78	0.0031	2.33
Q8WNV7	DHRS4	dehydrogenase/reductase SDR family member 4	8	34.08	0.0002	3.12
Q29214	RPLP0	60S acidic ribosomal protein P0	7	32.90	0.0131	2.73
F2Z565	SLC25A5	ADP/ATP translocase 1	7	29.50	0.0289	2.55
A0A5G2QPK4	AK3	GTP:AMP phosphotransferase AK3, mitochondrial	7	32.94	0.0041	1.95
P00889	CS	citrate synthase, mitochondrial	7	17.47	0.0012	2.04
A0A481CYL0	ACO1	cytoplasmic aconitate hydratase	7	38.73	0.0050	1.53
A0A480NVF8	KRT8	keratin, type II cytoskeletal 8	7	21.79	0.0040	7.38
A0A480JGI4	NCL	nucleolin	7	23.69	0.0001	4.71
A0A480I6I0	COL14A1	collagen alpha-1(XIV) chain isoform X1	7	23.53	0.0052	2.02

A0A8D1GJ40	LOC100153899	serpin A3-8	7	22.59	0.0214	4.16
P28491	CALR	calreticulin	7	24.68	0.0100	1.84
P00336	LDHB	L-lactate dehydrogenase B chain isoform X1	7	46.22	0.0069	1.71
A0A480XPR9	BPHL	valacyclovir hydrolase precursor	7	32.52	0.0094	1.80
F1RUE3	ALDH5A1	succinate-semialdehyde dehydrogenase	7	32.15	0.0044	1.92
A0A8D0MNR5	PECR	peroxisomal trans-2-enoyl-CoA reductase	6	42.25	0.0012	3.23
I3LUP6	NPM1	nucleophosmin	6	30.66	0.0000	3.17
A0A8D1NUI3	HOGA1	4-hydroxy-2-oxoglutarate aldolase, mitochondrial	6	21.93	0.0027	6.38
Q4GWZ2	RPSA	40S ribosomal protein S2	6	20.05	0.0118	2.03
P09623	DLD	dihydrolipoyl dehydrogenase, mitochondrial	6	30.59	0.0006	3.23
A0A4X1UNE1	PPA2	inorganic pyrophosphatase 2, mitochondrial	6	20.20	0.0286	1.73
A0A480RBI9	RPL7	60S ribosomal protein L7	6	22.54	0.0046	2.87
I3LLY3	ACTN1	alpha-actinin-1	6	31.30	0.0075	1.84
A0A2C9F3C0	GUSB	beta-glucuronidase precursor	6	31.85	0.0485	1.63
A0A4X1SVF9	POSTN	periostin	6	25.99	0.0044	2.85
A0A4X1W1U6	RPS4X	40S ribosomal protein S4, X isoform	6	14.98	0.0009	2.16
Q6PLK3	MAOB	amine oxidase [flavin-containing] B	6	18.58	0.0122	3.21
A0A480J4N2	C4	complement C4-A precursor	6	22.04	0.0118	2.47
P00348	HADH	hydroxyacyl-coenzyme A dehydrogenase, mitochondrial	6	33.60	0.0319	1.57
Q29550	CES1	liver carboxylesterase	6	25.54	0.0050	1.91
A0A4X1SK65	RPS3A	40S ribosomal protein S3a	6	20.64	0.0114	2.47
P00636	FBP1	fructose-1,6-bisphosphatase 1	6	28.59	0.0239	1.90
A0A8D1ILU6	PDIA4	protein disulfide-isomerase	6	38.83	0.0075	1.81
Q29387	EEF1G	elongation factor 1-gamma	6	30.22	0.0087	3.61
P80147	ABAT	4-aminobutyrate aminotransferase, mitochondrial	6	28.76	0.0193	2.02
A0A4X1UEN5	HSPA5	78 kDa glucose-regulated protein	6	22.07	0.0315	2.11
P28768	SOD2	superoxide dismutase [Mn], mitochondrial	6	34.21	0.0197	1.86
A0A8D0X3H3	ACAD8	isobutyryl-CoA dehydrogenase, mitochondrial	6	22.23	0.0052	2.18
Q29024	DDX39B	spliceosome RNA helicase DDX39B	5	20.28	0.0002	2.53
F1SKG5	GK	glycerol kinase	5	16.88	0.0052	1.62
A0A287BKR2	SHMT2	serine hydroxymethyltransferase	5	18.10	0.0003	2.37
A0A4X1U498	HNRNPA2B1	heterogeneous nuclear ribonucleoproteins A2/B1	5	23.35	0.0002	4.84
A0A4X1UTE2	HNRNPK	heterogeneous nuclear ribonucleoprotein K isoform X1	5	21.42	0.0017	2.07
O19069	SUCLG1	succinate-CoA ligase [ADP/GDP-forming] subunit alpha, mitochondrial	5	18.68	0.0001	2.30
A0A287B177	UGT1A6	UDP glucuronosyltransferase 1 family, polypeptide A6	5	16.14	0.0092	2.92
A0A4X1TIY7	HSPB6	heat shock protein beta-6	5	20.77	0.0006	8.10
A0A4X1W965	GLDC	glycine dehydrogenase (decarboxylating), mitochondrial	5	25.06	0.0003	3.86
A0A8D1ICB8	GART	trifunctional purine biosynthetic protein adenosine-3	5	20.74	0.0316	1.94
A0A287A3Q5	KARS1	lysine tRNA ligase isoform X1	5	15.99	0.0008	1.86
P63246	RACK1	receptor of activated protein C kinase 1	5	25.21	0.0262	1.92
P26042	MSN	moesin isoform X1	5	18.73	0.0128	2.14

F1RYS9	STBD1	starch-binding domain-containing protein 1	5	20.28	0.0017	2.31
I3L5K3	HSD17B11	estradiol 17-beta-dehydrogenase 11	5	16.63	0.0246	1.79
Q6WSP6	HSPE1	10 kDa heat shock protein, mitochondrial	5	24.75	0.0030	2.03
A0A8D1S7G0	LOC100523213	immunoglobulin lambda-like polypeptide 5	5	40.00	0.0029	1.93
P00795	CTSD	cathepsin D	5	21.24	0.0002	1.81
A0A8D1PXU5	MSRA	mitochondrial peptide methionine sulfoxide reductase	5	25.10	0.0010	3.28
Q29092	HSP90B1	endoplasmin	5	14.98	0.0064	1.89
A0A4X1SUW3	PRDX3	thioredoxin-dependent peroxide reductase, mitochondrial	5	18.11	0.0027	3.44
A0A8W4F715	NIPSNAP1	protein NipSnap homolog 1	5	27.34	0.0001	2.76
P26044	RDX	radixin	5	21.61	0.0001	2.00
F1SHU0	HIBADH	3-hydroxyisobutyrate dehydrogenase, mitochondrial	5	42.92	0.0077	1.83
A0A480YXQ8	AASS	alpha-aminoadipic semialdehyde synthase, mitochondrial	5	24.92	0.0033	2.29
Q29099	PTBP1	polypyrimidine tract-binding protein 1	4	17.48	0.0015	2.51
K9J6J2	STOM	erythrocyte band 7 integral membrane protein	4	12.57	0.0046	3.20
Q0QF01	SDHA	succinate dehydrogenase [ubiquinone] flavoprotein subunit, mitochondrial isoform X1	4	15.76	0.0082	2.64
A0A8D0YSE2	SNX5	sorting nexin-5	4	12.32	0.0005	1.92
Q8MI68	MMUT	methylmalonyl-CoA mutase, mitochondrial precursor	4	13.24	0.0307	1.84
A0A5G2QHU0	COPA	coatomer subunit alpha	4	12.76	0.0201	2.55
Q9MZ16	VDAC1	voltage-dependent anion-selective channel protein 1	4	12.91	0.0237	4.89
A0A480YTG3	CALU	calumenin isoform X2	4	14.53	0.0018	2.41
I3LP78	RPL9	60S ribosomal protein L9 isoform X1	4	15.58	0.0368	12.75
A0A8D0VQ42	ES1	ES1 protein homolog, mitochondrial	4	16.33	0.0030	4.02
Q764M5	STAT1	signal transducer and activator of transcription 1	4	11.90	0.0426	2.70
A0A5G2R8A9	CPT2	carnitine O-palmitoyltransferase 2, mitochondrial	4	12.82	0.0204	8.64
A0A286ZZT0	NNT	NAD(P) transhydrogenase, mitochondrial	4	14.10	0.0319	47.44
F1RWI8	SCRN2	secernin-2	4	19.44	0.0495	2.49
Q29375	RPL7A	60S ribosomal protein L7a	4	13.39	0.0001	5.81
A0A480Y2E3	KNG1	kininogen-1	4	15.24	0.0026	2.21
A0A4X1VN86	GANAB	neutral alpha-glucosidase AB isoform X1	4	15.31	0.0109	2.73
A0A8D0THT1	SPTBN1	spectrin beta chain, non-erythrocytic 1	4	18.49	0.0000	6.35
A0A480KFK8	GCAT	2-amino-3-ketobutyrate coenzyme A ligase, mitochondrial isoform X7	4	16.91	0.0074	10.30
D2SW95	COPB1	coatomer subunit beta	4	17.79	0.0010	8.69
Q29593	TALDO1	transaldolase	4	15.63	0.0038	1.68
Q9BX68	HINT2	adenosine 5'-monophosphoramidase HINT2 isoform 1 precursor	4	20.33	0.0007	2.08
Q01105	SET	protein SET	4	18.59	0.0001	2.24
F1SER1	MAT1A	S-adenosylmethionine synthase isoform type-1	4	13.66	0.0291	1.63
L8B0S2	IGHG	IgG heavy chain precursor	4	39.63	0.0081	1.65
A0A4X1VUH0	ECI1	enoyl-CoA delta isomerase 1, mitochondrial	4	11.85	0.0154	1.72
F1RUE0	ACOT13	acyl-coenzyme A thioesterase 13	4	18.01	0.0036	4.33

Q95342	RPL18	60S ribosomal protein L18	4	14.42	0.0097	2.39
A0A8D1JSH0	ECH1	delta(3,5)-Delta(2,4)-dienoyl-CoA isomerase, mitochondrial	4	13.37	0.0040	2.21
A0A4X1SP78	ARCN1	coatomer subunit delta	4	14.20	0.0065	1.64
A0A287B6Y1	RPL30	60S ribosomal protein L30	4	19.73	0.0032	3.01
A0A4X1W9D1	RPS8	40S ribosomal protein S8	4	13.94	0.0015	2.58
P00258	FDX1	adrenodoxin, mitochondrial precursor	4	13.55	0.0040	2.43
Q29187	RPL4	60S ribosomal protein L4	4	12.69	0.0016	3.14
A0A4X1W4Q9	HNRNPA1	heterogeneous nuclear ribonucleoprotein A1	4	18.15	0.0017	1.56
I3L9C8	ILK	integrin-linked protein kinase	3	10.92	0.0106	2.66
A0A5G2R6M7	SERPINB9	serpin B9	3	13.81	0.0304	2.22
Q29308	RPS19	40S ribosomal protein S19	3	7.44	0.0220	3.34
Q95307	RPL13A	60S ribosomal protein L13a	3	7.38	0.0049	3.31
A0A4X1UXS0	ADHFE1	hydroxyacid-oxoacid transhydrogenase, mitochondrial	3	11.69	0.0103	2.17
A0A480MCK5	HNRNPF	heterogeneous nuclear ribonucleoprotein F	3	16.06	0.0021	2.43
A0A8D1PHA0	RPL19	60S ribosomal protein L19	3	9.59	0.0013	6.76
Q95276	RPL5	60S ribosomal protein L5	3	13.71	0.0003	5.32
Q29293	RPL30	60S ribosomal protein L3	3	10.64	0.0114	2.45
A0A8D1QNX0	BDH1	D-beta-hydroxybutyrate dehydrogenase, mitochondrial	3	21.60	0.0061	7.16
A0A287A9Y6	RPS7	40S ribosomal protein S7	3	8.08	0.0032	3.91
Q29201	RPS9	40S ribosomal protein S9	3	6.91	0.0035	4.15
A0A480UCK1	ALDH16A1	aldehyde dehydrogenase family 16 member A1	3	11.08	0.0009	2.08
A0A8D0N0I3	SEPTIN7	septin-7	3	9.72	0.0210	3.36
A0A8D1LUZ8	PON3	serum paraoxonase/lactonase 3 precursor	3	16.53	0.0081	24.60
P53027	RPL10A	60S ribosomal protein L10a	3	7.47	0.0034	5.57
A0A5G2R3H5	USP5	ubiquitin carboxyl-terminal hydrolase 5 isoform X1	3	11.84	0.0339	2.11
A0A480QHS7	CORO1A	coronin-1A	3	10.49	0.0343	2.42
F1S4P6	EIF3A	eukaryotic translation initiation factor 3 subunit A	3	10.72	0.0227	2.30
A0A287AZG0	ARHGAP1	rho GTPase-activating protein 1 isoform X1	3	8.44	0.0409	27.79
A0A8D1GR47	CYP3A46	cytochrome P450 family 3 subfamily A member 46	3	9.22	0.0199	34.31
A0A480TM87	RARS1	arginine-tRNA ligase, cytoplasmic	3	10.75	0.0083	7.19
A0A4X1VIM6	GLYCTK	glycerate kinase	3	13.57	0.0022	4.02
A0A4X1SU77	AMT	aminomethyltransferase, mitochondrial	3	10.63	0.0001	3.44
P48819	VTN	vitronectin precursor	3	11.09	0.0003	2.93
A0A480JHQ3	EML4	microtubule-associated protein 4 isoform X6	3	10.17	0.0322	3.32
I3L920	CORO1C	coronin-1C	3	7.83	0.0278	2.48
A0A4X1SNP1	RPL27A	60S ribosomal protein L27a	3	8.84	0.0019	3.17
A0A287BJN5	LOC100518644	glycine N-acyltransferase-like isoform X1	3	11.36	0.0002	1.88
P79273	ACADS	short-chain specific acyl-CoA dehydrogenase, mitochondrial	3	17.96	0.0036	2.43
A0A8D0YLX5	RPS18	40S ribosomal protein S18	3	7.70	0.0004	3.07
A0A481DHP5	RPS25	40S ribosomal protein S25	3	7.80	0.0012	2.34
I3LC84	SULT1C4	sulfotransferase 1C4	3	8.99	0.0059	3.45

P14477	FGB	fibrinogen beta chain precursor	3	15.05	0.0442	1.78
A0A8D0IY26	HMGCL	hydroxymethylglutaryl-CoA lyase, mitochondrial	3	11.86	0.0417	1.58
A0A4X1SG32	LOC100155267	histone H2B type 2-F	3	9.76	0.0025	6.86
A0A8D1RQX0	HSD17B13	17-beta-hydroxysteroid dehydrogenase 13 isoform X1	3	12.52	0.0408	2.14
A0A2C9F366	HSPB1	heat shock protein beta-1	3	25.90	0.0074	3.08
A0A287AJ40	GLYAL	glycine N-phenylacetyltransferase	3	20.21	0.0003	2.54
A0A287BJ05	SEPTIN2	septin-2	3	10.67	0.0062	2.13
A0A480X9E7	SEC13	protein SEC13 homolog	3	10.62	0.0125	2.68
A0A4X1SG83	RPL24	60S ribosomal protein L24	3	7.44	0.0000	16.13
I3L650	CALD1	caldesmon isoform X1	3	11.24	0.0101	1.80
Q9N0F1	DLST	dihydrolipoyllysine-residue succinyltransferase component of 2-oxoglutarate dehydrogenase complex, mitochondrial	3	17.53	0.0026	3.32
A0A8D0INC8	PLS3	plastin-3	3	16.05	0.0073	1.64
A0A5C8H4Q8	GALK	galactokinase	3	9.40	0.0322	28.41
A0A8D1NWL4	SEPTIN11	septin-11 isoform X1	3	9.37	0.0015	2.21
A0A4X1VFZ3	RPS23	40S ribosomal protein S23	3	12.23	0.0022	2.34
A0A4X1T7J4	ANP32A	acidic leucine-rich nuclear phosphoprotein 32A	3	13.49	0.0000	2.56
A0A480VE77	RPL8	60S ribosomal protein L8	3	7.73	0.0077	2.10
A0A480SME4	RPSA	40S ribosomal protein SA	3	14.91	0.0026	2.70
A0A8D1ABH5	TXNDC5	thioredoxin domain-containing protein 5	3	9.29	0.0403	1.66
A0A8D0JDR5	HNRNPA3	heterogeneous nuclear ribonucleoprotein A3	3	16.16	0.0039	2.28
A0A4X1TFR5	RPS13	40S ribosomal protein S13	3	7.13	0.0015	2.32
A0A0K1TQS8	UDPGST2B31	UDP-glucuronosyltransferase 2B31 precursor	2	8.09	0.0412	6.10
A0A480TXX4	EIF3	eukaryotic translation initiation factor 3 subunit F	2	7.93	0.0124	2.15
A0A287AEH0	PSMD7	26S proteasome non-ATPase regulatory subunit 7	2	13.96	0.0018	4.06
F1RW98	RSU1	ras suppressor protein 1 isoform 1	2	7.97	0.0334	2.40
A0A480VHC2	RPL37A	60S ribosomal protein L37a isoform X1	2	7.79	0.0088	3.89
A0A8D1JF65	ARPC1B	actin-related protein 2/3 complex subunit 1B	2	4.63	0.0188	2.37
A0A8D0J214	VPS29	vacuolar protein sorting-associated protein 29 isoform X1	2	4.67	0.0126	2.13
A0A287BRL8	MARCKS	myristoylated alanine-rich C-kinase substrate	2	9.93	0.0039	1.96
O62786	SLC2A2	solute carrier family 2, glucose transporter member 2	2	5.96	0.0012	12.32
A0A480R697	MATR3	matrin-3 isoform X1	2	7.39	0.0101	11.02
P63221	RPS21	40S ribosomal protein S21	2	5.43	0.0042	5.11
F1SI10	RPL10L	60S ribosomal protein L10-like	2	5.24	0.0346	2.67
A0A4X1VRZ2	RPL36	60S ribosomal protein L36	2	5.81	0.0028	4.26
P47788	THOP1	thimet oligopeptidase	2	5.64	0.0213	1.78
A0A0K1TQS8	UDPGST2B31	UDP-glucuronosyltransferase 2B31	2	6.55	0.0307	2.22
A0A286ZLH8	RPL35A	60S ribosomal protein L35a	2	4.92	0.0068	2.77
A0A480SM35	AHSA1	activator of 90 kDa heat shock protein ATPase homolog 1	2	5.79	0.0035	2.58
A0A8D0LY87	SEC23A	protein transport protein Sec23A	2	5.26	0.0022	1.69

A0A4X1VTJ6	TIMM13	mitochondrial import inner membrane translocase subunit Tim13	2	7.37	0.0500	18.33
A0A480XFY9	CLYBL	citrate lyase subunit beta-like protein, mitochondrial	2	7.72	0.0004	2.86
A0A5G2QLY5	SND1	staphylococcal nuclease domain-containing protein 1	2	6.67	0.0004	214.34
F1SH89	CHDH	choline dehydrogenase, mitochondrial	2	7.96	0.0272	5.21
P14632	LTF	lactotransferrin	2	7.89	0.0064	3.69
A0A4X1TBE8	GSTM3	glutathione S-transferase Mu 3	2	7.25	0.0323	2.62
A0A286ZQD0	HIBCH	3-hydroxyisobutyryl-CoA hydrolase, mitochondrial	2	7.81	0.0385	2.33
K9IWC7	TRIM25	E3 ubiquitin/ISG15 ligase TRIM25	2	8.20	0.0184	33.38
A0A480TXZ6	EIF3I	eukaryotic translation initiation factor 3 subunit I	2	7.46	0.0012	4.28
A0A287ACX4	FKBP2	peptidyl-prolyl cis-trans isomerase FKBP2	2	11.68	0.0331	2.60
A0A287B773	MCCC2	methylcrotonoyl-CoA carboxylase beta chain, mitochondrial	2	8.24	0.0003	9.53
Q29381	DDOST	dolichyl-diphosphooligosaccharide-protein glycosyltransferase 48 kDa subunit	2	6.58	0.0193	7.46
A0A0K1TQN0	GSTA2	glutathione S-transferase	2	7.03	0.0320	29.29
Q9GMB0	RPN1	dolichyl-diphosphooligosaccharide--protein glycosyltransferase subunit 1 precursor	2	7.33	0.0000	Infinity
P05207	PRKAR2A	cAMP-dependent protein kinase type II-alpha regulatory subunit	2	9.36	0.0004	5.57
P55931	ETFDH	electron transfer flavoprotein-ubiquinone oxidoreductase, mitochondrial	2	6.60	0.0011	158.81
A0A4X1SW13	LDHD	D-lactate dehydrogenase, mitochondrial	2	6.67	0.0097	29.53
A0A4X1UIK2	CYP27A1	sterol 26-hydroxylase, mitochondrial	2	7.93	0.0327	20.65
A0A8D1JE65	TUFM	elongation factor Tu, mitochondrial	2	8.07	0.0041	9.25
Q6QRN9	SLC25A6	ADP/ATP translocase 3	2	4.96	0.0115	31.43
I3L7Y1	RPL26L1	60S ribosomal protein L26	2	5.65	0.0095	3.84
A0A480X4F2	VPS35	vacuolar protein sorting-associated protein 35	2	4.01	0.0217	8.53
A0A8X9AEI6	ATP5F1C	ATP synthase subunit gamma, mitochondrial	2	6.13	0.0138	16.22
A0A287B231	UBQLN1	ubiquilin-1	2	11.78	0.0037	3.29
A0A480L247	AFMID	kynurenine formamidase	2	11.40	0.0358	4.19
A0A480QMH4	HNRNP	heterogeneous nuclear ribonucleoprotein A/B	2	4.47	0.0374	11.01
A0A480T023	C8A	complement component C8 alpha chain precursor	2	5.91	0.0419	2.57
A0A5G2QD80	DCTN2	dynactin subunit 2 isoform X1	2	4.39	0.0296	5.04
P16164	UOX	uricase precursor	2	5.45	0.0053	6.80
A0A287BQZ1	HAO2	2-Hydroxyacid oxidase 1	2	5.73	0.0045	1.59
A0A287APN0	CSRP1	cysteine and glycine-rich protein 1	2	11.45	0.0195	1.68
A0A4X1TUJ4	ARPC5	actin-related protein 2/3 complex subunit 5	2	8.37	0.0453	1.87
P27917	APOC3	apolipoprotein C-III	2	8.53	0.0019	7.79
P27485	RBP4	retinol-binding protein 4	2	5.79	0.0008	2.24
P62831	RPL23	60S ribosomal protein L23	2	8.64	0.0219	2.34
A0A287BAK0	RPS24	40S ribosomal protein S24	2	7.71	0.0062	2.32
A0A8D0HP58	PHYHD1	phytanoyl-CoA dioxygenase domain-containing protein 1	2	8.73	0.0029	17.28
A0A287BFY0	ALDOA	fructose-bisphosphate aldolase A	2	6.49	0.0005	2.01

A1E295	CATSB	cathepsin B	2	8.67	0.0089	2.64
Q28960	CBR1	carbonyl reductase [NADPH] 1	2	8.23	0.0353	1.53
A0A5G2QBQ4	PRDX4	peroxiredoxin-4	2	12.21	0.0188	2.01
A0A4X1TSU2	ALDOC	fructose-bisphosphate aldolase C	2	7.25	0.0347	2.66
Q29550	CES1	liver carboxylesterase precursor	2	8.72	0.0010	2.10
A0A8D1GJ40	LOC100153899	serpin A3-8	2	8.12	0.0005	1184.33
A0A480KNE9	ETHE1	persulfide dioxygenase ETHE1, mitochondrial	2	7.28	0.0038	2.36
Q2YGT9	RPL6	60S ribosomal protein L6	2	5.05	0.0015	4.11
A0A8D0LHS2	RPS11	40S ribosomal protein S11	2	4.75	0.0062	2.51
A0A8D0Q4H0	RPL27A	60S ribosomal protein L27	2	4.84	0.0037	3.99
A0A480V1M7	AP2B1	AP-2 complex subunit beta	2	8.87	0.0070	1.85
Q6QAP7	RPS17	40S ribosomal protein S17	2	6.57	0.0004	2.47
A0A8D1AFD3	ANP32B	acidic leucine-rich nuclear phosphoprotein 32B	2	4.94	0.0002	7.45
A0A4X1SYE3	GCSH	glycine cleavage system H protein, mitochondrial	2	8.49	0.0001	2.77
P62895	CYC	cytochrome c	2	10.70	0.0160	1.83
F2Z4Z1	YWHAG	14-3-3 protein gamma	2	12.22	0.0023	2.05
A0A4X1WB36	ECHDC2	enoyl-CoA hydratase domain-containing protein 2, mitochondrial isoform X5	2	10.16	0.0009	3.21
A0A8D0HZX2	YWHAQ	14-3-3 protein theta	2	9.32	0.0063	1.71
D5KRL7	RPS10	40S ribosomal protein S10	2	7.53	0.0103	1.96
P67985	RPL22	60S ribosomal protein L22	2	5.95	0.0229	2.14
P49933	NPG4	protegrin-4	2	7.00	0.0244	2.84
P46405	RPS12	40S ribosomal protein S12	2	6.92	0.0013	2.74
A0PFK7	CAPZB	F-actin-capping protein subunit beta	2	7.89	0.0005	1.77

[1] Accession number in the UniProt database; [2] Matching peptides for each protein, used for quantitation; [3] SEQUEST score; The presence of the notation "Infinity" in the fold change column indicates the presence of the protein only in the PG samples.
