# Supplementary material for: Label-Free Quantitative Analysis of Pig Liver Proteome after Hepatitis E Virus Infection
Source: Viruses. 2024 Mar 6;16(3):408. doi: 10.3390/v16030408 (PMC10976091; doi:10.3390/v16030408)
Supplement: Supplementary file 1 [file viruses-16-00408-s001.zip › Supplementary_Table_S2_viruses-2770250.pdf]

Supplementary Table S2. Proteins identified with greater abundance in samples obtained from pigs that resulted negative to the ELISA evaluation (NG) following label-free MS/MS analysis (Progenesis QI for proteomics).

UniProt ID[1]	Gene name	Protein Description	Peptide[2]	Score[3]	Anova (p)	Fold change
Q58GK8	FASN	fatty acid synthase	24	124.48	0.0194	1.82
Q95332	BHMT	betaine-homocysteine S-methyltransferase 1	19	140.67	0.0003	1.78
L7TEV7	AOX1	aldehyde oxidase	18	91.88	0.0072	2.06
F1S1E7	DPYS	dihydropyrimidinase isoform X1	17	108.90	0.0015	1.86
A0A4X1VFQ7	LOC110255172	acyl-coenzyme A amino acid N-acyltransferase 2-like	17	93.37	0.0078	1.75
A0A480ST43	ASS1	argininosuccinate synthase	16	97.76	0.0015	1.66
F1S3H8	RDH8	retinol dehydrogenase 1	16	76.58	0.0276	1.54
A0A4X1UZ96	UPB1	beta-ureidopropionase	16	94.43	0.0086	1.98
P23687	PREP	prolyl endopeptidase	16	71.89	0.0139	2.11
Q28943	DPYD	dihydropyrimidine dehydrogenase [NADP(+)] precursor	15	67.95	0.0017	1.98
Q06AA3	RGN	regucalcin	14	94.49	0.0034	1.71
A0A5G2QAG3	LAP3	cytosol aminopeptidase isoform X1	14	79.04	0.0016	1.78
P46410	GLUL	glutamine synthetase isoform X1	13	70.53	0.0008	3.78
A0A480R4L3	KYNU	kynureninase isoform X1	13	50.36	0.0008	2.24
A0A286ZZN9	GLMS	glutamine-fructose-6-phosphate aminotransferase 1	13	58.72	0.0000	2.19
P37111	ACY1	aminoacylase-1	12	86.53	0.0009	2.34
Q6Q2C2	EPHX2	bifunctional epoxide hydrolase 2	12	54.89	0.0174	1.66
A0A286ZRS0	GSS	glutathione synthetase	11	44.56	0.0071	1.91
P28839	LAP3	xaa-Pro dipeptidase	11	48.18	0.0023	2.64
A0A4X1TBE8	GSTM3	glutathione S-transferase	11	49.20	0.0023	1.72
I3L804	YARS1	tyrosine-tRNA ligase, cytoplasmic isoform X1	11	41.54	0.0005	2.35
I3LIM2	UGDH	UDP-glucose 6-dehydrogenase	11	45.24	0.0098	1.82
Q02110	HPD	4-hydroxyphenylpyruvate dioxygenase	11	55.49	0.0033	1.65
D2SW95	COPB1	coatomer subunit beta	11	40.14	0.0082	1.67
A0A8D0YFM9	GSR	glutathione reductase, mitochondrial	10	58.65	0.0093	1.98
A0A480NMC5	DPP3	dipeptidyl peptidase 3 isoform X1	10	53.31	0.0009	2.71
O97788	FABP4	fatty acid-binding protein	10	54.41	0.0011	1.67
A0A4X1TZD5	EEF1A	elongation factor 1-alpha 1	10	61.46	0.0008	2.28
P03974	VCP	transitional endoplasmic reticulum ATPase	10	45.68	0.0014	2.04
F1SNJ5	AKR1D1	3-oxo-5-beta-steroid 4-dehydrogenase isoform X1	10	52.58	0.0026	1.75
F1SIJ9	PSAT1	phosphoserine aminotransferase	10	59.02	0.0008	1.63
F1S9K5	EPRS1	bifunctional glutamate/proline--tRNA ligase isoform X1	9	41.69	0.0451	2.18
K9IVR7	WDR1	WD repeat-containing protein 1	9	67.88	0.0366	2.38
Q99028	COMT	catechol O-methyltransferase	9	78.39	0.0053	1.82
A0A480TVJ2	MAN2C1	alpha-mannosidase 2C1 isoform X2	9	38.14	0.0021	6.66
A0A8D0TR45	PTER	phosphotriesterase-related protein isoform X1	9	52.76	0.0000	1.99
Q7SIB7	PGK1	phosphoglycerate kinase 1	9	51.09	0.0007	1.60

A0A287BBJ7	ALDH33A	alcohol dehydrogenase class-3	9	45.44	0.0002	1.65
A0A480Y1M9	XPNPEP1	xaa-Pro aminopeptidase 1 isoform X1	9	34.78	0.0003	1.86
F1RUY9	CARNS1	carnosine synthase 1 isoform X1	9	38.58	0.0072	2.22
A0A287AWU9	ENOSF1	enolase superfamily member 1	9	48.55	0.0001	1.95
P10775	RNH1	ribonuclease inhibitor isoform X1	9	42.75	0.0011	2.11
F1RLI9	SHPK	sedoheptulokinase	8	48.63	0.0015	2.23
A0A8W4F7Z7	ABHD14B	protein ABHD14B	8	46.33	0.0379	1.63
A0A287BGW4	ALAD	delta-aminolevulinic acid dehydratase	8	43.06	0.0131	2.31
P17560	RENBP	N-acylglucosamine 2-epimerase	8	39.24	0.0037	2.77
A0A480UBN5	MTAP	S-methyl-5'-thioadenosine phosphorylase	8	37.02	0.0001	1.74
A0A481BBL3	ALDH8A1	aldehyde dehydrogenase family	8	33.72	0.0150	2.02
A0A287ABJ0	BHMT2	S-methylmethionine--homocysteine S-methyltransferase BHMT2	8	48.89	0.0000	1.58
P08059	GPI	glucose-6-phosphate isomerase	8	53.26	0.0025	2.43
A0A8D0QAZ7	BLMH	bleomycin hydrolase	8	36.83	0.0010	2.01
A0A287BBK0	DDAH1	N(G),N(G)-dimethylarginine dimethylaminohydrolase 1	8	39.94	0.0001	1.77
A0A480STU2	LHPP	phospholysine phosphohistidine inorganic pyrophosphate phosphatase	7	46.60	0.0001	2.71
A0A287BIW1	GALE	UDP-glucose 4-epimerase	7	33.62	0.0112	1.85
A0A4X1VDX2	SORD	sorbitol dehydrogenase	7	39.66	0.0080	1.64
O62839	CAT	catalase	7	57.81	0.0325	1.69
P51781	GST	glutathione S-transferase alpha M14	7	37.13	0.0464	1.54
A0A480RXC8	AARS	alanine-tRNA ligase, cytoplasmic	7	34.63	0.0002	3.04
A0A480JL46	GLOD4	glyoxalase domain-containing protein 4	7	27.13	0.0004	1.72
A0A480VV97	CMBL	carboxymethylenebutenolidase homolog	7	30.83	0.0096	2.04
A0A8W4FEH1	SEC14L3	SEC14-like protein 3 isoform X1	7	47.85	0.0144	1.90
F1S0Y8	ADH4	all-trans-retinol dehydrogenase [NAD(+)] ADH4	7	79.78	0.0026	2.73
A0A8D0XGZ4	LOC100737183	cytosolic beta-glucosidase	7	26.77	0.0104	1.91
A0A8D1SKK2	ASPDH	aspartate dehydrogenase domain-containing protein	7	27.26	0.0111	1.81
A0A8D1GC64	ADI1	acireductone dioxygenase	7	29.63	0.0002	2.26
A0A287AFJ9	GPHN	gephyrin	7	28.24	0.0001	2.15
A0A8D0YP96	SEC14L2	SEC14-like protein 2	7	40.27	0.0020	2.34
A0A287ASQ2	ADK	adenosine kinase	7	30.38	0.0083	1.83
F1RX12	GMDS	GDP-mannose 4,6 dehydratase	6	41.57	0.0016	1.68
A0A8D1TDT7	DIAPH1	protein diaphanous homolog 1 isoform X2	6	28.57	0.0000	3.17
P00355	GAPDH	glyceraldehyde-3-phosphate dehydrogenase	6	41.58	0.0140	18.99
A0A287ARN4	PGM1	phosphoglucomutase-1	6	37.77	0.0480	1.64
A0A5G2QYS6	GPD1L	glycerol-3-phosphate dehydrogenase 1-like protein	6	26.22	0.0032	2.31
F1SJ30	MPI	mannose-6-phosphate isomerase	6	26.04	0.0119	2.39
F1SQR7	HAL	histidine ammonia-lyase	6	29.63	0.0017	2.19
D0G776	LTA4H	leukotriene A-4 hydrolase	6	35.39	0.0322	2.15
I3LHC8	BDH2	3-hydroxybutyrate dehydrogenase type 2	6	26.59	0.0082	1.74
A0A286ZTC2	PBLD	phenazine biosynthesis-like domain-containing protein isoform X1	6	39.40	0.0011	1.66

A0A480PP18	PHGDH	D-3-phosphoglycerate dehydrogenase	6	22.34	0.0175	1.85
A0A8D1RFQ4	TF	serotransferrin precursor	6	24.64	0.0073	2.39
A0A8D0IHP8	GRHPR	glyoxylate reductase/hydroxypyruvate reductase	6	26.47	0.0018	1.96
A0A4X1VU35	GAMT	guanidinoacetate N-methyltransferase isoform X2	6	38.54	0.0011	2.38
P82125	AKR1E2	aldo-keto reductase family 1 member C1	6	28.42	0.0012	1.74
A0A4X1ULZ8	GCLC	glutamate-cysteine ligase catalytic subunit isoform X1	6	23.69	0.0180	1.67
O46658	CYP2D25	vitamin D(3) 25-hydroxylase	6	36.98	0.0354	2.22
A0A480ZBJ4	NDRG2	protein NDRG2 isoform X1	6	38.92	0.0053	2.38
A0A480V890	TTC38	tetratricopeptide repeat protein 38	6	26.90	0.0239	1.88
A0A8D0IC03	GMPPA	mannose-1-phosphate guanyltransferase alpha	5	19.38	0.0003	2.39
A0A8D1PJN0	TARS1	threonine--tRNA ligase, cytoplasmic	5	16.47	0.0001	2.16
A0A8D1MDA2	GAA	lysosomal alpha-glucosidase	5	19.92	0.0069	2.41
A0A286ZPY1	AHNAK	neuroblast differentiation-associated protein AHNAK	5	20.47	0.0028	3.85
Q6QAR9	NPEPPS	puromycin-sensitive aminopeptidase	5	15.88	0.0001	2.51
F1RX88	LOC100152150	amine sulfotransferase-like isoform X1	5	26.83	0.0008	2.92
A0A8D1XB74	DDC	aromatic-L-amino-acid decarboxylase	5	22.37	0.0005	1.82
A0A480MFV9	KTN1	kinectin	5	17.19	0.0102	2.92
Q3S3F7	SULT2A1	sulfotransferase 2A1	5	19.17	0.0007	2.06
P12026	DBI	acyl-CoA-binding protein	5	34.33	0.0006	1.92
F1SBB0	OSBPL1A	oxysterol-binding protein-related protein 1	5	19.66	0.0054	1.75
A0A480JRT9	PSMD2	26S proteasome non-ATPase regulatory subunit 2	5	16.53	0.0389	1.51
A0A480KE10	NAGK	N-acetyl-D-glucosamine kinase	5	18.05	0.0151	1.87
A0A287B8R2	NUDC	nuclear migration protein nudC	5	16.39	0.0027	2.51
A0A5G2QFH3	KHK	ketohexokinase	5	41.10	0.0011	1.76
A0A480YJR0	AKR1C1	aldo-keto reductase family 1 member C1	5	33.28	0.0124	2.03
Q9TV69	DHDH	trans-1,2-dihydrobenzene-1,2-diol dehydrogenase	5	27.66	0.0104	1.70
F1SKY2	NIT2	omega-amidase NIT2 isoform X1	5	20.05	0.0076	1.79
P20304	IDH1	isocitrate dehydrogenase [NADP] cytoplasmic isoform X1	5	30.65	0.0324	1.71
D0G6X4	FDPS	farnesyl pyrophosphate synthase	5	33.38	0.0066	1.70
F1SUP1	AKR7A2	aflatoxin B1 aldehyde reductase member 2	5	24.41	0.0090	2.12
Q45FY6	HPRT1	hypoxanthine-guanine phosphoribosyltransferase	5	28.62	0.0023	1.98
Q9N1F5	GSTO1	glutathione S-transferase omega-1	5	28.58	0.0053	1.69
Q52I78	NAMPT	nicotinate phosphoribosyltransferase	5	31.34	0.0009	1.54
A0A480KQF0	CARKD	ATP-dependent (S)-NAD(P)H-hydrate dehydratase	5	16.09	0.0003	2.57
A0A4X1V3T5	PSMB5	proteasome subunit beta type-5	5	26.25	0.0004	2.43
A0A287BI79	PLS1	plastin-1	5	18.29	0.0154	2.36
A0A480NH91	ENO1	alpha-enolase isoform X1	5	31.91	0.0065	1.71
A0A480SKP2	CNDP2	cytosolic non-specific dipeptidase isoform X1	5	38.86	0.0060	1.66
F1SQ09	LUM	lumican precursor	5	23.10	0.0171	2.01

A0A5G2RBV3	UMPS	uridine 5'-monophosphate synthase	4	18.85	0.0006	2.70
A0A480IL99	PSMC2	26S proteasome regulatory subunit 7	4	15.85	0.0008	2.60
A0A480PM54	PRPSAP2	phosphoribosyl pyrophosphate synthase-associated protein 2 isoform X1	4	21.53	0.0034	1.95
F1SGL4	BBOX1	gamma-butyrobetaine dioxygenase	4	13.50	0.0061	5.07
P82125	AKR1E2	1,5-anhydro-D-fructose reductase isoform X1	4	16.27	0.0028	2.22
A0A287AA45	HNMT	histamine N-methyltransferase	4	16.26	0.0021	2.17
P54612	PPP2R1A	serine/threonine-protein phosphatase 2A 65 kDa regulatory subunit A alpha isoform	4	23.26	0.0257	1.71
A0A4X1WBL9	CCT4	T-complex protein 1 subunit delta	4	12.66	0.0029	2.19
D0G0C8	CCT2	T-complex protein 1 subunit beta	4	15.42	0.0042	Infinity
Q29081	PMVK	phosphomevalonate kinase	4	16.17	0.0059	3.43
I3LM71	IDO2	indoleamine 2,3-dioxygenase 2 isoform X1	4	17.21	0.0131	3.70
F2Z5J1	PSMC1	26S proteasome regulatory subunit 4	4	17.93	0.0156	1.71
A0A286ZI55	GPD1	glycerol-3-phosphate dehydrogenase [NAD(+)], cytoplasmic	4	27.55	0.0021	2.28
P79303	UGP	UTP-glucose-1-phosphate uridylyltransferase	4	27.17	0.0143	1.74
A1XQU1	PSMB7	proteasome subunit beta type-7	4	15.84	0.0091	2.77
A0A4X1SUI4	UFM1	ubiquitin-fold modifier 1	4	17.52	0.0074	2.03
Q9GJT2	ESD	S-formylglutathione hydrolase	4	23.89	0.0336	2.19
A0A286ZTA4	ALDOB	fructose-bisphosphate aldolase B	4	48.68	0.0475	1.52
A0A8D1L3X4	PRDX5	peroxiredoxin-5, mitochondrial	4	24.71	0.0003	3.65
A0A4X1WCV0	PPM1A	protein phosphatase 1A	4	16.00	0.0016	4.38
P50447	SERPINA1	alpha-1-antitrypsin	4	18.35	0.0006	1.75
Q8SQ65	CYP2C49	cytochrome P450 2C18	4	14.87	0.0080	4.00
A0A4X1TW19	PON1	serum paraoxonase/arylesterase 1	4	17.80	0.0079	2.35
P0C5I2	GMPPB	mannose-1-phosphate guanyltransferase beta	4	26.40	0.0000	1.94
A0A287AHB5	CSAD	cysteine sulfinic acid decarboxylase	4	16.46	0.0373	2.47
A0A287B5Q7	PSMD8	26S proteasome non-ATPase regulatory subunit 1	3	13.70	0.0028	1.84
P60982	DSTN	destrin	3	11.69	0.0003	2.12
A0A8D0NNP7	ACTR2	actin-related protein 2	3	13.93	0.0251	2.46
F1RXA7	PSMD3	26S proteasome non-ATPase regulatory subunit 3	3	9.42	0.0019	2.11
B3VMR0	ATIC	bifunctional purine biosynthesis protein ATIC	3	15.89	0.0078	3.26
O77591	IMPA1	inositol monophosphatase 1	3	11.50	0.0107	4.31
P67937	TPM4	tropomyosin alpha-4 chain isoform X1	3	9.67	0.0396	1.50
A0A4X1UPA0	TPK1	thiamin pyrophosphokinase 1	3	22.47	0.0009	2.31
A0A480SBA5	PSMD13	26S proteasome non-ATPase regulatory subunit 13	3	13.48	0.0081	1.64
A0A4X1UPZ8	AACS	acetoacetyl-CoA synthetase	3	14.49	0.0096	2.67
A0A1B2TT60	CYP3A22	cytochrome P450, subfamily IIIA, polypeptide 22	3	13.07	0.0002	25.37
A0A4X1TKU2	COPS4	COP9 signalosome complex subunit 4	3	13.71	0.0024	2.24
A0A8D0T416	DPYSL2	dihydropyrimidinase-related protein 2	3	11.51	0.0404	Infinity
A0A480U2R8	TIPRL	TIP41-like protein	3	14.49	0.0001	8.16
Q8SQ26	CRYL1	lambda-crystallin homolog	3	16.89	0.0388	2.09

A0A4X1W5L4	HSPG	basement membrane-specific heparan sulfate proteoglycan core protein	3	11.80	0.0005	20.36
A0A286ZTS1	L3HYPDH	trans-3-hydroxy-L-proline dehydratase	3	14.92	0.0000	4.24
A7TX81	COPS6	COP9 signalosome complex subunit 6	3	10.52	0.0004	2.15
P19205	APEH	acylamino-acid-releasing enzyme	3	12.41	0.0025	2.09
Q2EHH8	PPP1CA	serine/threonine-protein phosphatase PP1-alpha catalytic subunit	3	14.06	0.0015	3.40
A0A480VN60	SGTA	small glutamine-rich tetratricopeptide repeat-containing protein alpha isoform X1	3	9.85	0.0112	5.97
A0A4X1U7K3	ARPC1A	actin-related protein 2/3 complex subunit 1A	3	10.34	0.0014	2.31
A0A4X1UUG9	GALT	galactose-1-phosphate uridylyltransferase	3	17.02	0.0101	1.59
A0A480M761	GLRX	glutaredoxin-3	3	12.59	0.0010	3.84
A0A480IRT3	ASMTL	N-acetylserotonin O-methyltransferase-like protein isoform X1	3	10.24	0.0243	1.52
A0A8D1H6A2	UBE2L3	ubiquitin-conjugating enzyme E2 L3	3	12.46	0.0036	1.80
F2Z5C1	ANXA5	annexin A5	3	9.76	0.0020	3.79
A0A4X1UY49	PSMA4	proteasome subunit alpha type-4	3	13.18	0.0056	1.74
F1S850	AS3MT	arsenite methyltransferase isoform X1	3	14.07	0.0110	1.72
Q29384	PSMB4	proteasome subunit beta type-4	3	15.31	0.0238	2.38
A0A8D1J8M3	MVK	mevalonate kinase	3	13.40	0.0231	1.86
P52552	PRDX2	peroxiredoxin-2	3	16.06	0.0003	6.66
A0A8D0I393	HSD17B14	17-beta-hydroxysteroid dehydrogenase 14	3	23.39	0.0117	2.20
F1RJH8	ASL	argininosuccinate lyase	3	15.10	0.0077	1.75
Q8MJ30	QDPR	dihydropteridine reductase	3	12.43	0.0004	1.89
Q5S1S4	CA3	carbonic anhydrase 3	3	23.63	0.0218	2.19
F2Z5K2	PSMA5	proteasome subunit alpha type-5	3	14.77	0.0031	4.08
Q6Q7J2	GDI2	rab GDP dissociation inhibitor beta	3	15.90	0.0125	2.08
A0A287BM02	HPF1	ADP-ribosylation factor 1	3	13.27	0.0458	1.51
A0A4X1UL20	PSMA2	proteasome subunit alpha type-2	3	15.72	0.0005	2.12
A0A287A8X4	PRMT5	protein arginine N-methyltransferase 5	3	10.45	0.0000	3.69
I3LJW2	FGG	fibrinogen gamma chain	3	13.92	0.0476	3.13
Q5G6V9	CFL2	cofilin-2	3	13.17	0.0001	2.99
P83662	ACMSD	2-amino-3-carboxymuconate-6-semialdehyde decarboxylase	3	10.87	0.0193	3.57
F2Z5J9	RAB11B	ras-related protein Rab-11B	3	25.75	0.0016	2.85
A0A480MAC3	ARH3	poly(ADP-ribose) glycohydrolase ARH3	3	13.96	0.0035	2.48
A0A4X1VPX6	PMM2	phosphomannomutase 2	3	8.25	0.0136	2.02
P80895	PCMT1	protein-L-isoaspartate(D-aspartate) O-methyltransferase	3	16.92	0.0284	2.17
A0A8D0TL68	GMPR2	GMP reductase 2 isoform X1	3	17.00	0.0001	1.70
A0A287BPP9	PSMB1	proteasome subunit beta type-1	3	10.64	0.0227	1.99
A0A4X1T630	HGD	homogentisate 1,2-dioxygenase	3	15.91	0.0072	64.13
F1RTV1	PPAT	amidophosphoribosyltransferase	3	8.82	0.0105	2.53
A0A8D2A136	NMRK1	nicotinamide riboside kinase 1	3	11.20	0.0001	1.97
A0A480Y306	GMPR	GMP reductase 1	3	12.94	0.0044	2.22
A0A8D1KUM5	PUDP	pseudouridine-5'-phosphatase	2	7.52	0.0019	1.96

A0A8D1ET23	FN1	fibronectin	2	9.76	0.0011	102.35
A0A286ZHZ3	TSN	translin	2	13.07	0.0001	1.89
Q06AV1	NNMT	nicotinamide N-methyltransferase	2	9.24	0.0404	Infinity
A0A8D0LLR0	MTPN	myotrophin	2	14.43	0.0007	48.62
A0A4X1VGT1	OTUB1	ubiquitin thioesterase OTUB1	2	8.01	0.0014	3.30
F1RWJ5	KPNB1	importin subunit beta-1	2	13.52	0.0096	2.18
A0A287B9M9	EIF4G1	eukaryotic translation initiation factor 4 gamma 1	2	5.77	0.0009	3.72
A0A287ABP7	COPS3	COP9 signalosome complex subunit 3	2	6.78	0.0001	2.16
Q0PIT9	NAXE	NAD(P)H-hydrate epimerase precursor	2	5.96	0.0397	1.80
Q02038	NLN	neurolysin, mitochondrial	2	8.64	0.0001	2.81
F1RGD9	HARS1	histidine--tRNA ligase, cytoplasmic	2	8.14	0.0000	2.60
P81693	ACP1	low molecular weight phosphotyrosine protein phosphatase	2	14.49	0.0000	1.69
A0A480I1S7	FGGY	FGGY carbohydrate kinase domain-containing protein isoform X1	2	8.81	0.0136	3.10
I3LAT6	IARS1	isoleucine--tRNA ligase, cytoplasmic isoform X1	2	7.31	0.0119	19.46
A0A8D1KJM0	LOC110257570	TRPM8 channel-associated factor 2-like	2	7.24	0.0159	15.18
A0A480VYA5	NUDT9	ADP-ribose pyrophosphatase, mitochondrial isoform X1	2	6.44	0.0171	12.31
A0A4X1UWL1	PFN1	profilin-1	2	10.52	0.0368	3.37
A0A287A7V3	HEXA	beta-hexosaminidase subunit alpha	2	7.00	0.0000	Infinity
A0A8D0M3L7	DPP2	dipeptidyl peptidase 2 isoform X1	2	9.82	0.0004	1979.33
A0A287AAG0	UAP1L1	UDP-N-acetylhexosamine pyrophosphorylase-like protein 1	2	7.32	0.0032	2.23
A0A4X1U5P7	RAB18	ras-related protein Rab-18	2	6.89	0.0040	2.56
A0A8D0LZI3	CSN2	COP9 signalosome complex subunit 2	2	4.55	0.0026	3.76
A0A8D0WIM3	GCK	glucokinase	2	7.81	0.0447	2.84
A1YIZ1	SEPHS2	selenide, water dikinase 2	2	10.02	0.0043	3.63
A0A8D1CE83	TTC36	tetratricopeptide repeat protein 36	2	7.67	0.0042	2.02
A0A287A021	NUTF2	nuclear transport factor 2	2	15.01	0.0009	2.60
A0A8D1B579	PPP1R7	protein phosphatase 1 regulatory subunit 7	2	8.23	0.0007	2.06
Q9GJX5	CYP4A21	taurochenodeoxycholic 6 alpha-hydroxylase	2	7.52	0.0432	2.31
A0A481D833	FTH1	ferritin heavy chain isoform X1	2	8.20	0.0227	1.70
F1SB63	TCP1	T-complex protein 1 subunit alpha	2	5.65	0.0017	1.88
A0A8W4FQB3	IDI1	isopentenyl-diphosphate Delta-isomerase 1	2	9.80	0.0426	23.67
A0A8D1YQU9	MTHFR	methylenetetrahydrofolate reductase	2	6.07	0.0043	50.86
Q9GKX6	GALM	galactose mutarotase	2	11.02	0.0220	2.83
A0A4X1TBE8	GSTM3	glutathione S-transferase	2	7.20	0.0077	6.11
A0A8D0J2P5	ACAT	acetyl-CoA acetyltransferase, cytosolic	2	15.87	0.0037	1.57
A0A5K1UWY1	GSTZ1	maleylacetoacetate isomerase	2	15.04	0.0080	3.07
A0A286ZV95	TMED10	transmembrane emp24 domain-containing protein 10	2	6.35	0.0089	1.65
F1SSL6	PSMA3	proteasome subunit alpha type-3	2	7.89	0.0009	2.03
A0A286ZN52	PSMB3	proteasome subunit beta type-3	2	10.24	0.0086	3.11
P14332	PGD	6-phosphogluconate dehydrogenase, decarboxylating	2	17.61	0.0169	31.57

F1RG31	HAGH	hydroxyacylglutathione hydrolase, mitochondrial isoform X1	2	18.01	0.0031	2.59
A0A480ZCU6	SPR	sepiapterin reductase	2	9.98	0.0190	2.08
Q2EN76	NME2	nucleoside diphosphate kinase B	2	9.39	0.0006	2.71
I3LCU9	CARHSP1	calcium-regulated heat-stable protein 1	2	6.69	0.0019	1.81
P19133	FTL	ferritin light chain	2	13.96	0.0085	1.96
A0A4X1UDT9	IAH1	isoamyl acetate-hydrolyzing esterase 1 homolog isoform X1	2	11.52	0.0011	1.84
Q9GL51	PAFAH1B1	platelet-activating factor acetylhydrolase IB subunit alpha	2	9.57	0.0014	2.04
I3LCC2	MARS1	methionine--tRNA ligase, cytoplasmic	2	10.43	0.0000	Infinity
F1SK86	FKBP4	peptidyl-prolyl cis-trans isomerase FKBP4	2	8.23	0.0000	Infinity
A0A480NVY9	ASRGL1	isoaspartyl peptidase/L-asparaginase	2	11.06	0.0027	2.31
A0A4X1VSC7	UFC1	ubiquitin-fold modifier-conjugating enzyme 1	2	8.69	0.0004	4.15
F1S1X3	NARS1	asparagine--tRNA ligase, cytoplasmic	2	7.16	0.0129	2.72
B3LDT2	CTSZ	cathepsin Z	2	8.41	0.0167	1.96
F1SGH9	ACOX2	peroxisomal acyl-coenzyme A oxidase 2	2	15.14	0.0268	3.11
A0A4X1UVQ7	GCHFR	GTP cyclohydrolase 1 feedback regulatory protein	2	10.60	0.0001	3.26
A0A255VEC9	coaBC	phosphopantothenate--cysteine ligase isoform X1	2	5.42	0.0232	4.49
A0A4X1SSI5	GNPDA1	glucosamine-6-phosphate isomerase 1	2	7.05	0.0017	1.77
A0A5G2QI52	CPPED1	serine/threonine-protein phosphatase CPPED1	2	7.79	0.0055	1.74
F1STK8	C9H11orf54	ester hydrolase C11orf54 homolog	2	10.95	0.0025	5.90
A0A8D1E909	FKBP1A	peptidyl-prolyl cis-trans isomerase FKBP1A	2	8.85	0.0013	2.68
Q007T2	CDC42	cell division control protein 42 homolog	2	6.31	0.0234	1.79
A0A5G2QX22	UBA5	ubiquitin-like modifier-activating enzyme 5	2	5.91	0.0414	1.74
A0A287BPG3	NAGA	alpha-N-acetylgalactosaminidase precursor	2	8.44	0.0126	1.98
Q9GMA6	SERPINA3-2	alpha-1-antichymotrypsin 2	2	7.99	0.0021	90.48

[1] Accession number in the UniProt database. [2] Matching peptides for each protein, used for quantitation. [3] SEQUEST score. The presence of the notation "Infinity" in the fold change column indicates the presence of the protein only in the NG samples.
