# Supplementary material for: Label-Free Quantitative Analysis of Pig Liver Proteome after Hepatitis E Virus Infection
Source: Viruses. 2024 Mar 6;16(3):408. doi: 10.3390/v16030408 (PMC10976091; doi:10.3390/v16030408)
Supplement: Supplementary file 1 [file viruses-16-00408-s001.zip › Supplementary_Table_S3_viruses-2770250.pdf]

Supplementary Table S3. Pathway Enrichment Analysis performed on differentially expressed proteins by using the web server SRplot.

Pathway Description	pvalue	p.adjust	qvalue	geneID	Count
Carbon metabolism	2,59897E-28	6,15956E-26	4,59607E-26	CPS1, GLUD1, PC, PCCA, IDH2, PCCB, ACAT1, SUCLG2, MDH2, ACADS, CS, ACO1, DLD, FBP1, SHMT2, SUCLG1, GLDC, SDHA, MMUT, TALDO1, GLYCTK, AMT, DLST, HIBCH, HAO2, ALDOA, ALDOC, GCSH, RGN, PSAT1, PGK1, GPI, CAT, GAPDH, PHGDH, IDH1, ENO1, ESD, ALDOB, GCK, PGD	41
Ribosome	7,46256E-22	8,84314E-20	6,59848E-20	RPL26L1, RPS3, RPLP0, RPSA, RPL7, RPS4X, RPS3A, RPL9, RPL7A, RPL18, RPL30, RPS8, RPL4, RPS19, RPL13A, RPL19, RPL5, RPS7, RPS9, RPL10A, RPL27A, RPS18, RPS25, RPL24, RPS23, RPL8, RPS13, RPL37A, RPS21, RPL10L, RPL36, RPL35A, RPL23, RPS24, RPL6, RPS11, RPS17, RPS10, RPL22, RPS12	40
Valine, leucine and isoleucine degradation	3,48906E-21	2,75636E-19	2,05671E-19	ALDH2, PCCA, PCCB, AGXT2, ACAA2, EHHADH, ACADSB, HADHA, ACAT1, IVD, ACADM, HMGCS2, HADHB, ACADS, DLD, HADH, ABAT, ACAD8, HIBADH, MMUT, HMGCL, HIBCH, MCCC2, AOX1, AACS	25
Glyoxylate and dicarboxylate metabolism	4,51301E-19	2,67396E-17	1,99522E-17	PCCA, PCCB, ACAT1, MDH2, CS, ACO1, HOGA1, DLD, SHMT2, GLDC, MMUT, GLYCTK, AMT, AFMID, HAO2, GCSH, GLUL, CAT, GRHPR	19
Coronavirus disease	3,13475E-16	1,48587E-14	1,10871E-14	RPL26L1, RPS3, RPLP0, RPSA, RPL7, RPS4X, RPS3A, RPL9, STAT1, RPL7A, RPL18, RPL30, RPS8, RPL4, RPS19, RPL13A, RPL19, RPL5, RPS7, RPS9, RPL10A, RPL27A, RPS18, RPS25, FGB, RPL24, RPS23, RPL8, RPS13, RPL37A, RPS21, RPL10L, RPL36, RPL35A, C8A, RPL23, RPS24, RPL6, RPS11, RPS17, RPS10, RPL22, RPS12, FGG	44
Glycine, serine and threonine metabolism	1,75568E-14	6,93492E-13	5,17463E-13	DMGDH, SARDH, AGXT2, GATM, DLD, MAOB, SHMT2, GLDC, GCAT, GLYCTK, AMT, CHDH, GCSH, BHMT, PSAT1, PHGDH, GRHPR, GAMT	18
Biosynthesis of cofactors	2,42675E-14	8,21629E-13	6,13074E-13	ALDH2, NADK2, AK2, MTHFD1, AK3, DLD, GUSB, SHMT2, UGT1A6, MAT1A, AFMID, RGN, KYNU, GSS, UGDH, HPD, PSAT1, EPRS1, ALAD, ASPDH, GPHN, MPI, GCLC, GMPPA, UMPS, IDO2, GMPPB, TPK1, PMM2, SPR, NME2	31
Biosynthesis of amino acids	8,43167E-14	2,49788E-12	1,86384E-12	CPS1, PC, IDH2, OTC, CS, ACO1, SHMT2, TALDO1, MAT1A, ALDOA, ALDOC, ASS1, GLUL, ACY1, PSAT1, PGK1, GAPDH, PHGDH, IDH1, ENO1, ALDOB, ASL	22
Tryptophan metabolism	2,6632E-12	7,0131E-11	5,23296E-11	ALDH2, EHHADH, HADHA, ACAT1, GCDH, DLD, MAOB, HADH, DLST, AFMID, AOX1, KYNU, ALDH8A1, CAT, DDC, IDO2, ACMSD	17
Propanoate metabolism	1,08885E-11	2,58058E-10	1,92555E-10	ACSS3, PCCA, PCCB, EHHADH, HADHA, SUCLG2, ACADS, LDHB, LDHB, DLD, ABAT, SUCLG1, MMUT, HIBCH	14
Proteasome	2,377E-11	5,12134E-10	3,82139E-10	PSMD7, PSMD2, PSMB5, PSMC2, PSMC1, PSMB7, PSMD8, PSMD3, PSMD13, PSMA4, PSMB4, PSMA5, PSMA2, PSMB1, PSMA3, PSMB3	16
Fatty acid degradation	9,66808E-11	1,90945E-09	1,42477E-09	ALDH2, ACAA2, EHHADH, ACADSB, ACSL1, HADHA, ACAT1, ACADM, HADHB, GCDH, ACADS, HADH, CPT2, ECI1, ADH4	15
Glycolysis / Gluconeogenesis	4,00754E-10	7,30605E-09	5,45155E-09	ALDH2, PCK1, LDHB, LDHB, DLD, FBP1, ALDOA, ALDOC, PGK1, GPI, ADH4, GAPDH, PGM1, ENO1, ALDOB, GCK, GALM	17
Citrate cycle (TCA cycle)	7,46056E-10	1,26297E-08	9,42387E-09	PC, PCK1, IDH2, SUCLG2, MDH2, CS, ACO1, DLD, SUCLG1, SDHA, DLST, IDH1	12
Amino sugar and nucleotide sugar metabolism	1,09443E-09	1,7292E-08	1,29027E-08	UGDH, RENBP, GPI, GALE, GMDS, PGM1, MPI, GMPPA, NAGK, GMPPB, GALT, PMM2, UAP1L1, GCK, GNPDA1	15
Butanoate metabolism	1,75386E-09	2,5979E-08	1,93848E-08	EHHADH, HADHA, ACAT1, HMGCS2, ACADS, ALDH5A1, HADH, ABAT, BDH1, HMGCL, AACS	11
Biosynthesis of nucleotide sugars	2,33795E-09	3,25938E-08	2,43205E-08	UGDH, GPI, GALE, GMDS, PGM1, MPI, GMPPA, NAGK, GMPPB, GALT, PMM2, UAP1L1, GCK	13
Fructose and mannose metabolism	2,89204E-09	3,80785E-08	2,8413E-08	FBP1, ALDOA, ALDOC, ENOSF1, SORD, GMDS, MPI, GMPPA, KHK, ALDOB, GMPPB, PMM2	12
beta-Alanine metabolism	4,3731E-09	5,45486E-08	4,07025E-08	ALDH2, EHHADH, HADHA, ACADS, ABAT, HIBCH, DPYS, UPB1, DPYD, CARNS1, CNDP2, CSAD	12
Peroxisome	1,36419E-08	1,61656E-07	1,20623E-07	IDH2, EHHADH, ACSL1, PRDX1, DHRS4, PECR, SOD2, ECH1, HMGCL, HAO2, LOC110255172, EPHX2, CAT, IDH1, PMVK, PRDX5, MVK, ACOX2	18
Cysteine and methionine metabolism	2,46221E-08	2,77879E-07	2,07344E-07	AGXT2, TST, MDH2, LDHB, LDHB, MAT1A, BHMT, GSS, PSAT1, MTAP, BHMT2, ADI1, PHGDH, GCLC	14
Alanine, aspartate and glutamate metabolism	3,82692E-08	4,12263E-07	3,07618E-07	CPS1, GLUD1, AGXT2, ALDH4A1, ALDH5A1, ABAT, ASS1, GLUL, NIT2, ASL, PPAT, ASRGL1	12
Pyruvate metabolism	1,28715E-07	1,32632E-06	9,89659E-07	PC, ALDH2, PCK1, ACAT1, MDH2, LDHB, LDHB, DLD, LDHD, ADH4, GRHPR, HAGH	12
Pentose phosphate pathway	1,5859E-07	1,56607E-06	1,16855E-06	FBP1, TALDO1, GLYCTK, ALDOA, ALDOC, RGN, GPI, PGM1, ALDOB, PGD	10
Fatty acid metabolism	5,08058E-07	4,81639E-06	3,59384E-06	ACAA2, EHHADH, ACADSB, ACSL1, HADHA, ACAT1, HSD17B8, ACADM, HADHB, ACADS, HADH, CPT2, FASN	13
Arginine biosynthesis	1,10135E-05	0,000100392	7,49097E-05	CPS1, GLUD1, OTC, ASS1, GLUL, ACY1, ASL	7
Spinocerebellar ataxia	1,16196E-05	0,000101995	7,61052E-05	SLC25A5, VDAC1, PSMD7, SLC25A6, PSMD2, PSMB5, PSMC2, PSMC1, PSMB7, PSMD8, PSMD3, PSMD13, PSMA4, PSMB4, PSMA5, PSMA2, PSMB1, PSMA3, PSMB3	19
PPAR signaling pathway	2,04098E-05	0,000172754	0,000128904	PCK1, EHHADH, ACSL1, ACADM, HMGCS2, GK, CPT2, ILK, CYP27A1, APOC3, FABP4, DBI, ACOX2	13
Arginine and proline metabolism	4,39425E-05	0,000359116	0,000267962	ALDH2, GATM, ALDH4A1, HOGA1, MAOB, LAP3, CARNS1, GAMT, CNDP2, L3HYPDH	10
Parkinson disease	6,37822E-05	0,000490694	0,00036614	ATP5F1B, ATP5F1A, SLC25A5, MAOB, HSPA5, SDHA, VDAC1, PSMD7, SLC25A6, ATP5F1C, PSMD2, PSMB5, PSMC2, PSMC1, PSMB7, PSMD8, PSMD3, PSMD13, UBE2L3, PSMA4, PSMB4, PSMA5, PSMA2, PSMB1, PSMA3, PSMB3	26

Amyotrophic lateral sclerosis	6,47182E-05	0,000490694	0,00036614	ATP5F1B, ATP5F1A, HSPA5, HNRNPA2B1, SDHA, VDAC1, HNRNPA1, SEC13, HNRNPA3, PSMD7, MATR3, ATP5F1C, UBQLN1, DCTN2, VCP, CAT, PSMD2, PSMB5, PSMC2, PSMC1, PSMB7, PSMD8, PSMD3, PSMD13, PSMA4, PSMB4, PSMA5, PSMA2, PSMB1, PFN1, PSMA3, PSMB3	32
One carbon pool by folate	6,6254E-05	0,000490694	0,00036614	MTHFD1, SHMT2, GART, AMT, ATIC, MTHFR	6
Pentose and glucuronate interconversions	7,49766E-05	0,000538468	0,000401788	GUSB, UGT1A6, UGDH, SORD, DHDH, CRYL1, FGGY	7
Drug metabolism - other enzymes	8,8446E-05	0,000616521	0,000460029	GUSB, CES1, UGT1A6, GSTM3, DPYS, UPB1, DPYD, HPRT1, GSTO1, UMPS, NME2	11
Tyrosine metabolism	0,000124795	0,000845042	0,000630544	MAOB, AOX1, HPD, COMT, ADH4, DDC, HGD, GSTZ1	8
Prion disease	0,000220318	0,001450425	0,001082262	ATP5F1B, ATP5F1A, SLC25A5, HSPA5, SDHA, VDAC1, PSMD7, SLC25A6, ATP5F1C, C8A, PSMD2, PSMB5, PSMC2, PSMC1, PSMB7, PSMD8, PSMD3, PSMD13, PSMA4, PSMB4, PSMA5, PSMA2, PSMB1, PSMA3, PSMB3	25
Histidine metabolism	0,000230577	0,001476939	0,001102047	ALDH2, MAOB, CARNS1, HAL, CNDP2, HNMT	6
Huntington disease	0,000254274	0,001585866	0,001183324	ATP5F1B, CLTC, ATP5F1A, SLC25A5, SOD2, SDHA, VDAC1, PSMD7, SLC25A6, ATP5F1C, DCTN2, AP2B1, PSMD2, PSMB5, PSMC2, PSMC1, PSMB7, PSMD8, PSMD3, PSMD13, PSMA4, PSMB4, PSMA5, PSMA2, PSMB1, PSMA3, PSMB3	27
Lysine degradation	0,000279562	0,001698878	0,001267651	ALDH2, EHHADH, HADHA, ACAT1, GCDH, DLD, HADH, AASS, DLST, BBOX1	10
Bacterial invasion of epithelial cells	0,000362656	0,002148735	0,00160332	VCL, CLTC, ILK, SEPTIN2, SEPTIN11, ARPC1B, ARPC5, ACTR2, ARPC1A, FN1, CDC42	11
Glutathione metabolism	0,000584685	0,003379766	0,002521877	IDH2, GSTM3, LAP3, GSS, GSR, GCLC, IDH1, GSTO1, PGD	9
2-Oxocarboxylic acid metabolism	0,000715466	0,004037275	0,00301249	IDH2, CS, ACO1, ACY1, IDH1	5
Steroid hormone biosynthesis	0,001230385	0,006781424	0,00506009	HSD17B8, UGT1A6, CYP3A46, AKR1D1, COMT, CYP2D25, AKR1C1, CYP2C49, CYP3A22	9
Galactose metabolism	0,001648388	0,008878816	0,006625099	GALE, PGM1, GAA, GALT, GCK, GALM	6
Ferroptosis	0,001715596	0,009035473	0,006741992	ACSL1, GSS, TF, TF, GCLC, FTH1, FTL	7
Pantothenate and CoA biosynthesis	0,001912467	0,009643717	0,007195844	ALDH2, DPYS, UPB1, DPYD, CSAD	5
Terpenoid backbone biosynthesis	0,001912467	0,009643717	0,007195844	ACAT1, HMGCS2, FDPS, PMVK, MVK	5
Metabolism of xenobiotics by cytochrome P450	0,002856752	0,013647984	0,010183706	UGT1A6, GSTM3, CBR1, ADH4, SULT2A1, DHDH, AKR7A2, GSTO1	8
Chemical carcinogenesis - DNA adducts	0,002856752	0,013647984	0,010183706	UGT1A6, CYP3A46, GSTM3, CBR1, SULT2A1, GSTO1, CYP2C49, CYP3A22	8
Ascorbate and aldarate metabolism	0,002879322	0,013647984	0,010183706	ALDH2, GUSB, UGT1A6, RGN, UGDH	5
Purine metabolism	0,003111408	0,014458895	0,010788783	AK2, AK3, GART, UOX, ADK, PGM1, HPRT1, ATIC, GMPR2, PPAT, GMPR, NUDT9, NME2	13
Nicotinate and nicotinamide metabolism	0,003649597	0,01663374	0,012411585	NADK2, NNT, AOX1, ASPDH, NAMPT, NMRK1	6
Nitrogen metabolism	0,003824136	0,017100382	0,012759779	CPS1, GLUD1, GLUL, CA3	4
Primary bile acid biosynthesis	0,006013473	0,026392464	0,019693244	CYP27A1, LOC110255172, AKR1D1, ACOX2	4
Retinol metabolism	0,006995559	0,029606205	0,022091239	DHRS4, UGT1A6, CYP3A46, AOX1, RDH8, ADH4, CYP2C49, CYP3A22	8
Aminoacyl-tRNA biosynthesis	0,006995559	0,029606205	0,022091239	KARS1, RARS1, YARS1, EPRS1, TARS1, HARS1, IARS1, NARS1	8
Folate biosynthesis	0,009033677	0,037561076	0,028026919	CBR1, GPHN, AKR1C1, QDPR, SPR	5
HIF-1 signaling pathway	0,0167823	0,068575952	0,051169265	LDHB, LDHB, ALDOA, ALDOC, PGK1, GAPDH, TF, TF, ENO1, ALDOB	10
Nucleotide metabolism	0,022185206	0,088987257	0,066399552	AK2, AK3, ADK, HPRT1, ASMTL, GMPR2, GMPR, NME2	8
Fatty acid elongation	0,02252842	0,088987257	0,066399552	ACAA2, HADHA, HADHB, HADH	4
Thiamine metabolism	0,023384491	0,090315977	0,067391002	AK2, TPK1, ACP1	3
Drug metabolism - cytochrome P450	0,023626965	0,090315977	0,067391002	MAOB, UGT1A6, GSTM3, AOX1, ADH4, GSTO1	6
Pyrimidine metabolism	0,027822699	0,104666344	0,078098804	DPYS, UPB1, DPYD, UMPS, ASMTL, NME2	6
Chemical carcinogenesis - reactive oxygen species	0,031191637	0,115506529	0,086187417	ATP5F1B, ATP5F1A, SLC25A5, SOD2, SDHA, VDAC1, GSTM3, SLC25A6, ATP5F1C, CBR1, EPHX2, CAT, AKR1C1, GSTO1, AS3MT, ACP1	16
Regulation of actin cytoskeleton	0,03230005	0,117770951	0,087877059	VCL, ACTN1, MSN, RDX, KNG1, ARPC1B, C8A, ARPC5, DIAPH1, ACTR2, PPP1CA, ARPC1A, CFL2, FN1, PFN1, CDC42	16
Alzheimer disease	0,035421404	0,127195042	0,094909025	ATP5F1B, ATP5F1A, SLC25A5, SDHA, VDAC1, PSMD7, SLC25A6, ATP5F1C, GAPDH, PSMD2, PSMB5, PSMC2, PSMC1, PSMB7, PSMD8, PSMD3, PSMD13, PSMA4, PSMB4, PSMA5, PSMA2, PSMB1, PSMA3, PSMB3	24
Fatty acid biosynthesis	0,038205709	0,135145567	0,100841462	ACSL1, HSD17B8, FASN	3
Antifolate resistance	0,044506897	0,155119626	0,11574549	SHMT2, GART, ATIC, MTHFR	4
Pathways of neurodegeneration - multiple diseases	0,045249934	0,155423687	0,115972372	ATP5F1B, ATP5F1A, SLC25A5, HSPA5, SDHA, VDAC1, PSMD7, SLC25A6, ATP5F1C, DCTN2, VCP, CAT, PSMD2, PSMB5, PSMC2, PSMC1, PSMB7, PSMD8, PSMD3, PSMD13, UBE2L3, PSMA4, PSMB4, PSMA5, PSMA2, PSMB1, PSMA3, PSMB3	28
